# Two dimensional Conjugated Metal–Organic Frameworks with Multiple Redox‐Active Sites towards High‐Performance Sodium‐Ion Battery

**DOI:** 10.1002/advs.202503369

**Published:** 2025-04-25

**Authors:** Meiling Qi, Linqi Cheng, Xupeng Zhang, Yuzhao Guo, Xi Su, Xiaoxiao Sun, Yi Liu, Lei Wang, Heng‐Guo Wang, Long Chen

**Affiliations:** ^1^ Shenzhen Key Laboratory of Polymer Science and Technology Guangdong Research Center for Interfacial Engineering of Functional Materials College of Materials Science and Engineering Shenzhen University Shenzhen 518060 China; ^2^ College of Physics and Optoelectronic Engineering Shenzhen University Shenzhen 518060 China; ^3^ Key Laboratory of Polyoxometalate and Reticular Material Chemistry of Ministry of Education Faculty of Chemistry Northeast Normal University Changchun 130024 China; ^4^ State Key Laboratory of Supramolecular Structure and Materials College of Chemistry Jilin University Changchun 130012 China

**Keywords:** cathode, conjugated metal–organic frameworks, multiple redox‐active sites, Scholl reaction, sodium‐ion battery

## Abstract

Two dimensional (2D) conjugated metal–organic frameworks (2D *c*‐MOFs) have emerged as promising electroactive materials for energy storage owing to their high conductivity and large charge carrier mobility. However, their broader implementation is hindered by limitations in capacity and cycling stability, primarily due to the restricted density, diversity, and stability of the redox sites. In this study, a new 2D *c*‐MOF (Cu‐TTPQ) with multiple redox‐active sites that incorporated quinone and pyrazine functionalities as cathode materials for sodium‐ion batteries (SIBs) is developed. Notably, 2D layered Cu‐TTPQ with a rigid skeleton is directly synthesized from a flexible precursor ligand through in situ cyclodehydrogenation and coordination assembly. Two other contrastive 2D *c*‐MOF analogs (Cu‐TBPQ and Cu‐DDQP) sharing similar structural motifs with Cu‐TTPQ but featuring distinct conductivities and energy band characteristics are prepared for systematic investigation. By contrast, Cu‐TTPQ demonstrates a higher reversible capacity of 214.8 mAh g^−1^ at 0.05 A g^−1^, along with high cycling stability, showing impressive cyclability with minimal capacity decay even after 1800 cycles at 5.0 A g^−1^. This work elucidates the rationality of introducing multiple redox‐active sites to improve the overall performance of 2D *c*‐MOFs as cathode materials for SIBs.

## Graphical abstract

Three isoreticular Two dimensional (2D) conjugated metal–organic framework (2D *c*‐MOF) analogs with different redox‐active centers are synthesized as cathode materials for high‐performance sodium‐ion batteries. Notably, the [CuO_4_] and TTPQ (integrating both quinone and pyrazine structures) units within Cu‐TTPQ, serving as multiple redox‐active sites, facilitate impressive larger reversible capacity along with superior cyclability.

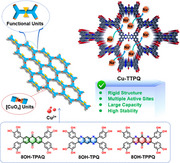

## 1 Introduction

As the global community grapples with resource scarcity and escalating prices, the diversified advancement of battery energy‐storage technologies has emerged as a pivotal strategy for mitigating the rising costs associated with commercial lithium‐ion batteries (LIBs).^[^ ^1^ ^]^ Among these alternatives, sodium‐ion batteries (SIBs) have garnered significant attention owing to their natural sodium abundant resources, cost‐effectiveness, enhanced safety, and competitive electrochemical performance.^[^ ^2^ ^]^ Notably, SIBs are particularly well suited for large‐scale energy storage applications, daily transportation vehicles, and other fields in which safety, cost, and temperature adaptability are essential. Nevertheless, the bulk intercalation of Na^+^ poses substantial challenges to the structural integrity of electrode materials, primarily because of the larger radius of Na^+^ (1.02 Å) than that of Li^+^ (0.76 Å).^[^ ^3^ ^]^ Therefore, the development of advanced electrode materials, especially cathodes for SIBs, has become a critical issue.

Two dimensional (2D) conjugated metal–organic frameworks (2D *c*‐MOFs) have emerged as promising platforms for electroactive energy storage.^[^ ^4^ ^]^ Their unique structural attributes confer several advantages: i) the robust frameworks ensure physicochemical stability in electrolytic environments; ii) the abundant pore structures provide adequate space and rapid transport channels for Na^+^, thereby maintaining structural integrity during the electrochemical process; iii) the tunability of periodically dispersed coordination centers (MX_4_, X = O, NH, etc.) and conjugated structures allows for facile modulation of intrinsic properties (such as conductivity, stacking mode, and energy band) and active sites, ultimately enhancing the electrochemical performance.^[^ ^5^, ^6^, ^7^ ^]^ Owing to these fascinating advantages, 2D *c*‐MOFs are promising electrode materials for secondary batteries.

**Scheme 1:**
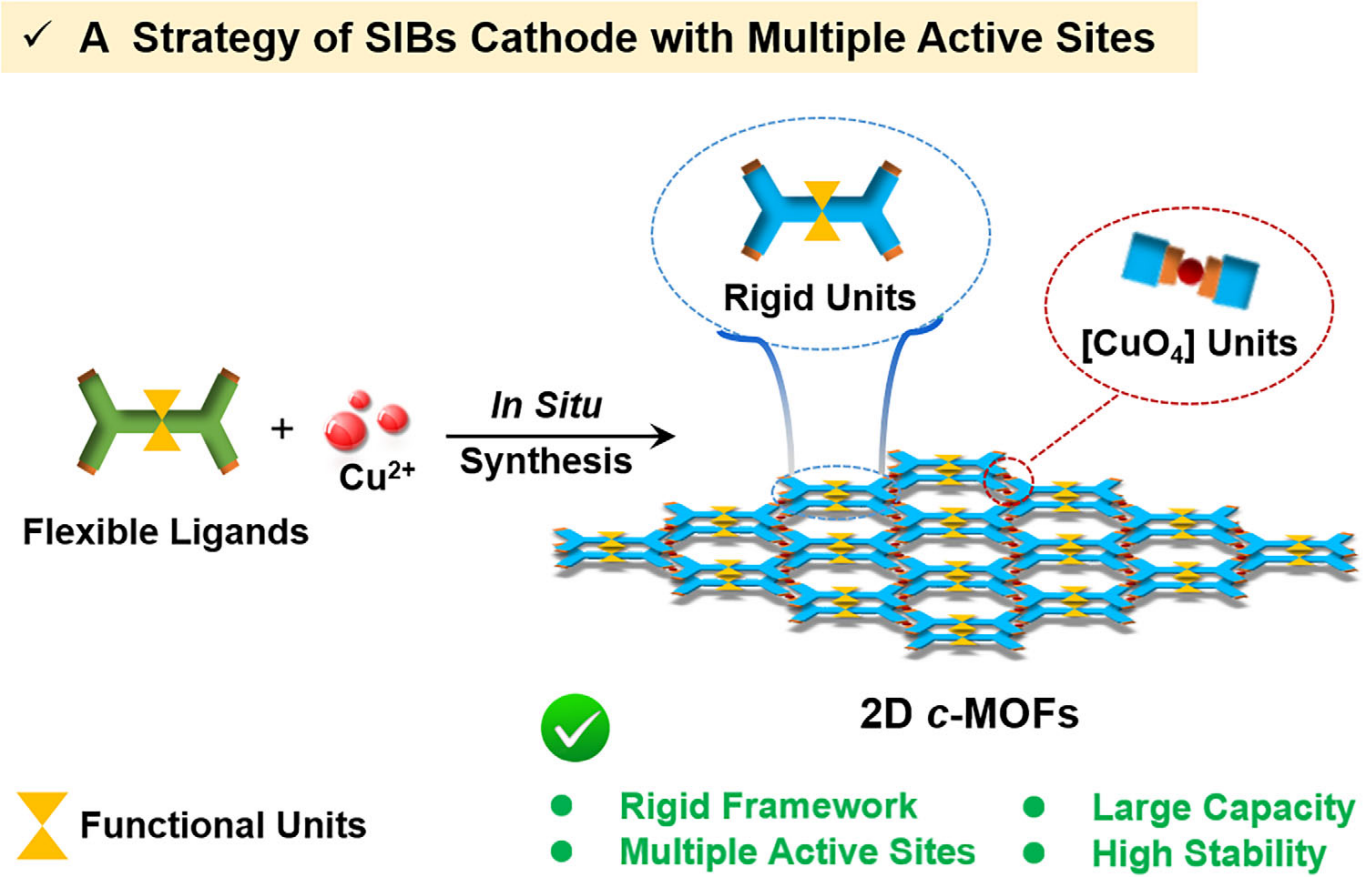
Illustration of a strategy for 2D *c*‐MOFs cathode materials with multiple active sites including rigid functional and [CuO_4_] units synthesized by flexible ligands.

Recent pioneering studies have highlighted the benefits of the enriched active sites and extended π‐conjugated skeletons in 2D *c*‐MOFs as cathode materials for SIBs.^[^ 6^b–d^ ^]^ Nevertheless, the capacity of most 2D *c*‐MOFs to achieve higher energy densities is often constrained by the [MX_4_] unit as the single redox site, which also influences their output voltages and cycling stability. Innovative design of functional organic ligands is crucial for enhancing the electrochemical properties of 2D *c*‐MOF electrode materials, which will promote the development of cathode materials from single to multiple active sites (Scheme 1). Specifically, functional organic ligands with extensive conjugation, narrow bandgaps, and high density of active sites are usually associated with broader voltage windows, increased specific capacities, and extended cycle lifetimes in secondary batteries.^[^ ^8^, ^9^, ^10^ ^]^ The lower LUMO (lowest unoccupied occupied molecular orbital) and higher HOMO (highest occupied molecular orbital) of the organic ligands possess broader operating voltage in batteries based on molecular orbital theory.^[^ ^6^, ^10^ ^]^ Therefore, there is an urgent need to rationally design molecules with multiple redox‐active centers with appropriate LUMO/HOMO levels to synthesize new 2D *c*‐MOFs. Additionally, the theoretical capacity of batteries is closely related to the density of active sites. Typically, the LUMO/HOMO levels could be altered by introducing electron‐withdrawing groups such as carbonyl (C═O) and pyrazine groups.^[^ ^1^, ^10^ ^]^ Furthermore, organic materials featuring C═O, pyrazine, amines, and other redox‐active groups have demonstrated exceptional electroactive properties.^[^ ^1^, ^2^, ^3^, ^11^ ^]^ Among these, quinone and pyrazine derivatives, featuring C═O and C═N unsaturated groups, stand out as cathode materials because of their high density of redox‐active centers.^[^ ^2^, ^11^ ^]^ These attributes render them promising candidates for metal‐ion batteries, offering substantial theoretical capacities and the potential for multi‐electron reactions. Nonetheless, poor solubility and tedious synthesis of large π‐conjugated rigid organic ligands have hindered the synthesis of highly crystalline 2D *c*‐MOFs.^[^ ^11^, ^12^ ^]^ Therefore, devising efficient synthetic strategies to address these challenges is essential for advancing the application of 2D *c*‐MOFs in SIBs.

In this study, a new π‐electron‐delocalized 2D *c*‐MOF (Cu‐TTPQ, TTPQ = tetrabenzo‐5,7,12,14‐tetraaza‐6,13‐pentacenequinone) with electron‐withdrawing quinone and pyrazine structures was designed to generate multiple redox‐active sites for SIBs. Additionally, analogous quinone‐based Cu‐TBPQ (TBPQ = tetrabenzo[a,c,l,n]pentacene‐10,21‐dione) and pyrazine‐based Cu‐DDQP (DDQP = dibenzo[a,c]dibenzo[5,6:7,8]quinoxalino[2,3‐i]phenazine), which share the partial structural characteristics of TTPQ, were synthesized for comparative analysis. These 2D *c*‐MOFs were synthesized in one pot directly from the corresponding flexible precursor ligands with respective quinone and pyrazine motifs. Notably, Cu‐TTPQ demonstrated an impressive reversible capacity of 214.8 mAh g^−1^ at 0.05 A g^−1^ and a high‐capacity retention rate of ≈99% (82.8 mAh g^−1^) after 1800 cycles at 5.0 A g^−1^. Moreover, the presence of multiple redox‐active sites and a two‐step reaction mechanism for Na^+^ storage were elucidated through density functional theory (DFT) calculations and experimental results. This work elucidates a facile strategy for enhancing the electrochemical performance of 2D *c*‐MOF cathode materials by incorporating multiple redox‐active sites into their skeletons.

## 2 Results and Discussion

**Figure 1:**
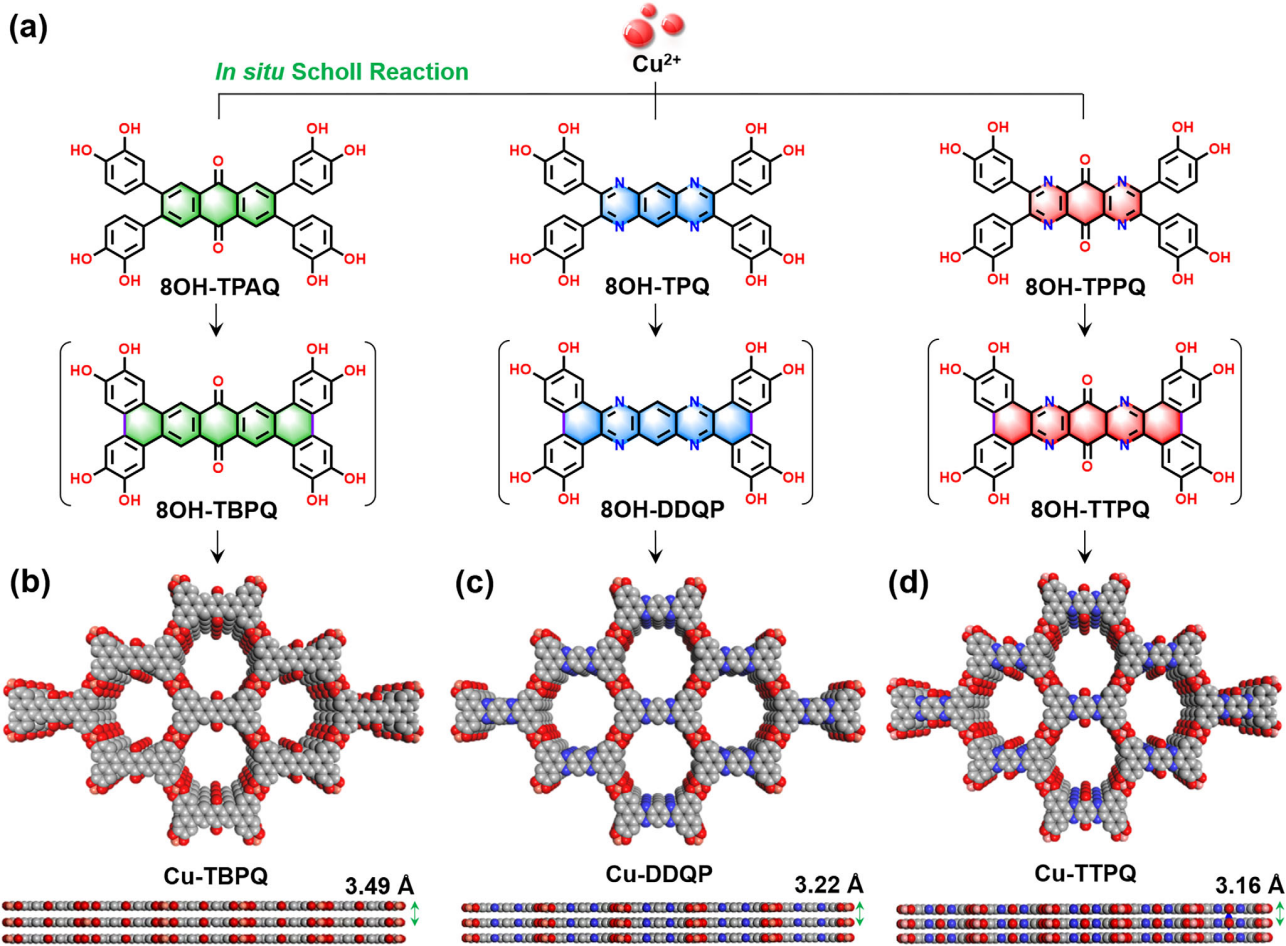
One‐pot synthesis schematic diagram of 2D *c*‐MOFs from flexible ligands via in situ Scholl reaction. The structures of a) flexible 8OH‐TPAQ, 8OH‐TPQ and 8OH‐TPPQ ligands, and their rigid 8OH‐TBPQ, 8OH‐DDQP, and 8OH‐TTPQ analogs, b) Cu‐TBPQ, c) Cu‐DDQP and d) Cu‐TTPQ.

All organic ligands and 2D *c*‐MOFs were synthesized and characterized according to procedures established in the literature (Schemes S1–S4 and Figures S1—S8, Supporting Information).^[^ ^13^ ^]^ Specifically, Cu‐TBPQ, Cu‐DDQP and Cu‐TTPQ were directly synthesized from their flexible precursors, including 2,3,6,7‐tetrakis(3,4‐dihydroxyphenyl)anthracene‐9,10‐dione (8OH‐TPAQ), 2,3,7,8‐tetrakis(3,4‐dihydroxyphenyl)pyrazino[2,3‐g]quinoxaline (8OH‐TPQ) and 2,3,7,8‐tetrakis(3,4‐dihydroxyphenyl)pyrazino[2,3‐g]quinoxaline‐5,10‐dione (8OH‐TPPQ) through an in situ Scholl reaction (**Figure** **1**) and coordination assembly.^[^ ^12a^ ^]^ As a proof of concept, the cyclodehydrogenation of the flexible precursors was further investigated (Figure S9, Supporting Information). However, in contrast to previous reports,^[^ ^12a^ ^]^ 8OH‐TPAQ, 8OH‐TPQ, and 8OH‐TPPQ exhibited varying degrees of partial cyclodehydrogenation when subjected to deionized water at 85 °C for three days (Figure S9b–d, Supporting Information, green lines). This discrepancy is probably due to their weakened ability to generate radical/onium cations, which serve as key intermediates for cyclodehydrogenation, stemming from the electron‐withdrawing effects of the centers.^[^ ^14a,b^ ^]^ Conversely, under the synthetic conditions for the 2D *c*‐MOFs, 8OH‐TPAQ, 8OH‐TPQ, and 8OH‐TPPQ were effectively converted into their planar counterparts, demonstrating that DMF and Cu^2+^ probably facilitated the transformation of flexible ligands into rigid  analogs (Figure S9b–d, Supporting Information, blue‐black lines). This may be attributed to the collaborative effect of Cu^2+^ as a Lewis acid catalyst and DMF as a cosolvent, whereas Cu^2+^ serves as an electron acceptor, which promotes dehydrogenation.^[^ ^14c^ ^]^

**Figure 2:**
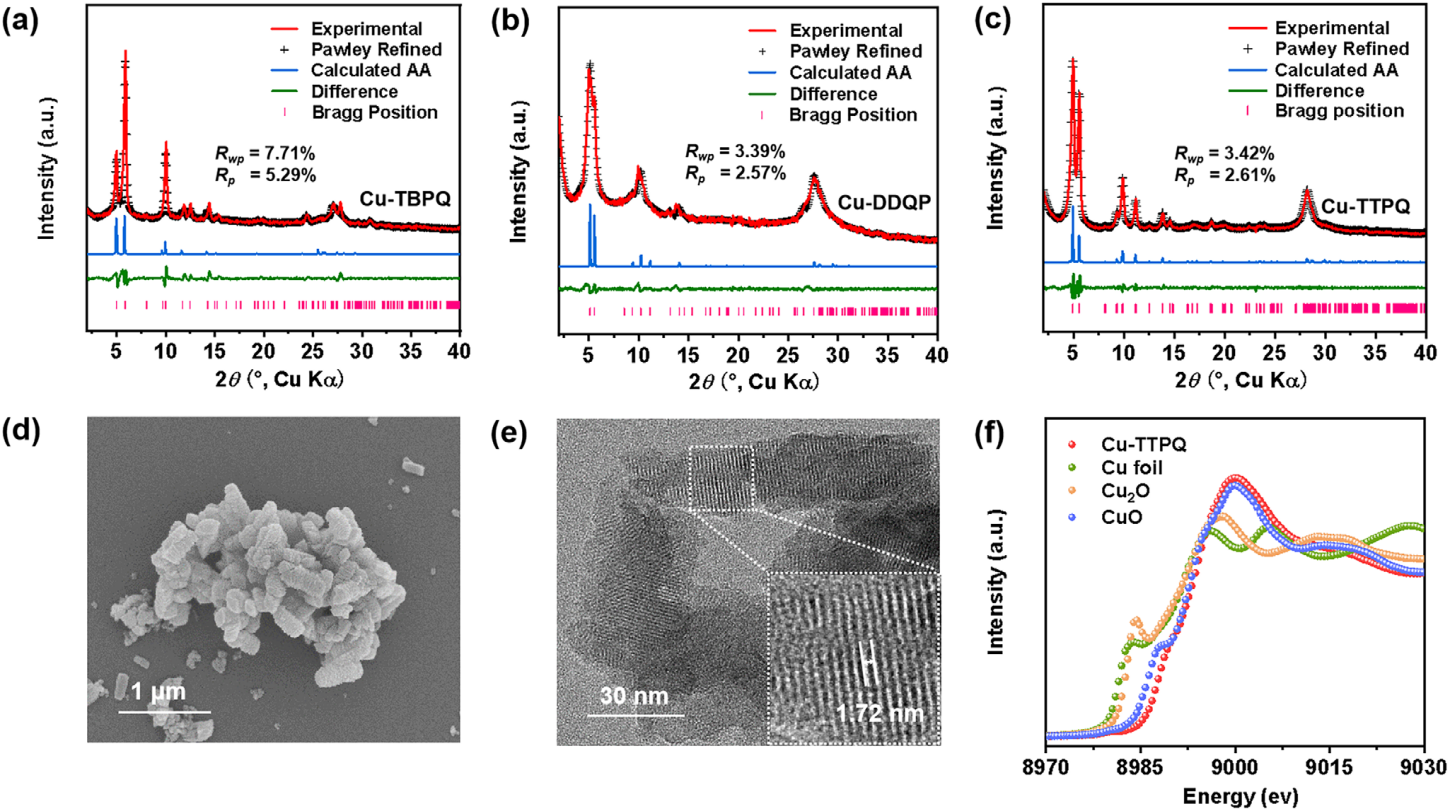
Structural characterization of Cu‐TBPQ, Cu‐DDQP, and Cu‐TTPQ. Experimental patterns (red line), Pawley refined patterns (black dots), the difference between experimental and refined patterns (green line), calculated pattern (blue line), and positions of reflections (pink dots) of (a) Cu‐TBPQ, (b) Cu‐DDQP and (c) Cu‐TTPQ. (d) SEM and (e) TEM images of Cu‐TTPQ. (f) Cu K‐edge of XANES for Cu‐TTPQ.

The structural characteristics of Cu‐TBPQ, Cu‐DDPQ and Cu‐TTPQ were further elucidated using Fourier transform infrared (FT‐IR) spectroscopy, ultraviolet‐visibble‐near‐infrared (UV–vis–NIR) spectroscopy, and powder X‐ray diffraction (PXRD) analysis (Figure 2a–c and Figures S10–S13, Supporting Information). As shown in Figure S10 (Supporting Information), the O─H stretching vibration band associated with 8OH‐TPPQ at ≈3350 cm^−1^ significantly weakened, accompanied by significant changes of C‐H bending vibration at 700–900 cm^−1^, indicating deprotonation and the formation of extended conjugated structures in Cu‐TTPQ.^[^ ^3^, ^6^ ^]^ The observation was corroborated by the broadened UV‐vis–NIR spectra (Figure S11, Supporting Information). To gain deeper structural insights, the experimental PXRD pattern of Cu‐TTPQ was compared with the simulated structures, revealing close alignment with the rhombic square lattice (*sql*)‐AA stacking model (Figure S12, Supporting Information). Pawley refinement afforded *PM_6_* space groups with unit cell parameters of *a* = 19.22 Å, *b* = 19.21 Å, and *c* = 3.16 Å, with *α* = *β* = 90° and *γ* = 111.2° (Figure 2c) with reasonable *Rwp* and *Rp* values of 3.42% and 2.61%, respectively. Additionally, morphological and crystalline assessments of Cu‐TTPQ were conducted using scanning electron microscopy (SEM) and transmission electron microscopy (TEM), revealing nanorod morphologies with discernible lattice spacings of ≈1.72, 1.57, 0.78, and 0.32 nm, corresponding to the *d*‐spacing (17.85, 15.73, 7.91 and 3.16 Å) of the (110), (020), (040), and (001) planes for Cu‐TTPQ (Figure 2d,e and Figure S15, Supporting Information). The Cu K‐edge X‐ray absorption near‐edge spectroscopy (XANES) spectrum confirmed the +2 oxidation state of the Cu centers, with the white‐line peak and Cu K‐edge energy aligned with those of CuO (Figure 2f). This was further validated by high‐resolution X‐ray photoemission spectroscopy (XPS), which identified a principal peak at 934.6 eV (Figure S19, Supporting Information). The coordination environment of the Cu centers was elucidated through Cu K‐edge extended X‐ray absorption fine structure (EXAFS) analysis, revealing a first coordination peak at 1.90 Å attributed to Cu─O bonding and a second peak at 2.66 Å corresponding to Cu···C bonding (Figure S16 and Table S1, Supporting Information). Furthermore, Cu‐TTPQ exhibited excellent chemical stability in various solvents and good thermal stability, with a weight loss of ≈5% at ≈385 °C after solvent loss (Figures S20 and S21, Supporting Information).

In contrast, Cu‐TBPQ and Cu‐DDQP featuring nanoparticle morphology were also synthesized and analyzed (Figures S13, S14, S17, and S18, Supporting Information).^[^ 11^b,c^ ^]^ The mixed oxidation state of Cu centers within two 2D *c*‐MOFs were confirmed by their XPS spectrums (Figures S17 and S18, Supporting Information). In addition, the experimental PXRD patterns and pore sizes of Cu‐TBPQ and Cu‐DDQP closely matched with their calculated patterns and the diameter (≈1.5 nm) of their hexagonal single‐pore models (Figure 2a,b; Figures S13 and S22, Supporting Information), indicating that they were successfully synthesized. These findings suggest that Cu‐TTPQ, Cu‐TBPQ, and Cu‐DDQP possess similar hexagonal single‐pore 2D framework structures stacked along the *c*‐axis, with comparable compositions, pore structures, and Brunauer‐Emmett‐Teller (BET) surface areas. However, the layer accumulation and *d*‐spacing exhibited significant variability because of the structural changes mediated by different organic ligands. The simulated structures of Cu‐TTPQ, Cu‐TBPQ, and Cu‐DDQP revealed the same overlapping AA stacking mode with the *d*‐spacing of 3.16, 3.21, and 3.49 Å, respectively (Figure 1b–d). The integration of both the electron‐withdrawing quinone and pyrazine structures in Cu‐TTPQ made the extended conjugated structure more rigid, facilitating interlayer π–π interactions.^[^ ^7^ ^]^

DFT calculations were performed to evaluate the molecular orbitals of the three conjugated ligands and assess the impact of the integrated quinone and pyrazine groups. As shown in Figure S24 (Supporting Information), the LUMO level of 8OH‐TTPQ, characterized by its electron‐withdrawing quinone and pyrazine structure, was found to be −2.73 eV, lower than that of 8OH‐TBPQ (−2.42 eV) and 8OH‐DDQP (−2.70 eV). This phenomenon can be ascribed to the diminished electron density, which results in a decrease in electron repulsion and an enhancement in electron delocalization.^[^ ^10a^ ^]^ Consequently, this alteration concurrently can influence the electrochemical characteristics of the Cu‐TTPQ cathode, facilitating its electron acceptance and resulting in an increased open circuit voltage.^[^ ^6c^ ^]^ This result was also further confirmed by the experimental LUMO‐HOMO energy gaps of the 2D *c*‐MOFs calculated through valence band (VB)‐XPS and UV–vis–NIR spectra (Figure S25 and Table S3, Supporting Information).^[^ ^6^, ^15^ ^]^ In addition, the introduction of quinone and pyrazine structures not only increased the discharge voltage but also served as additional active sites for ionic storage to enhance the specific capacity.^[^ ^8^, ^9^, ^10^, ^11^, ^16^ ^]^

**Figure 3:**
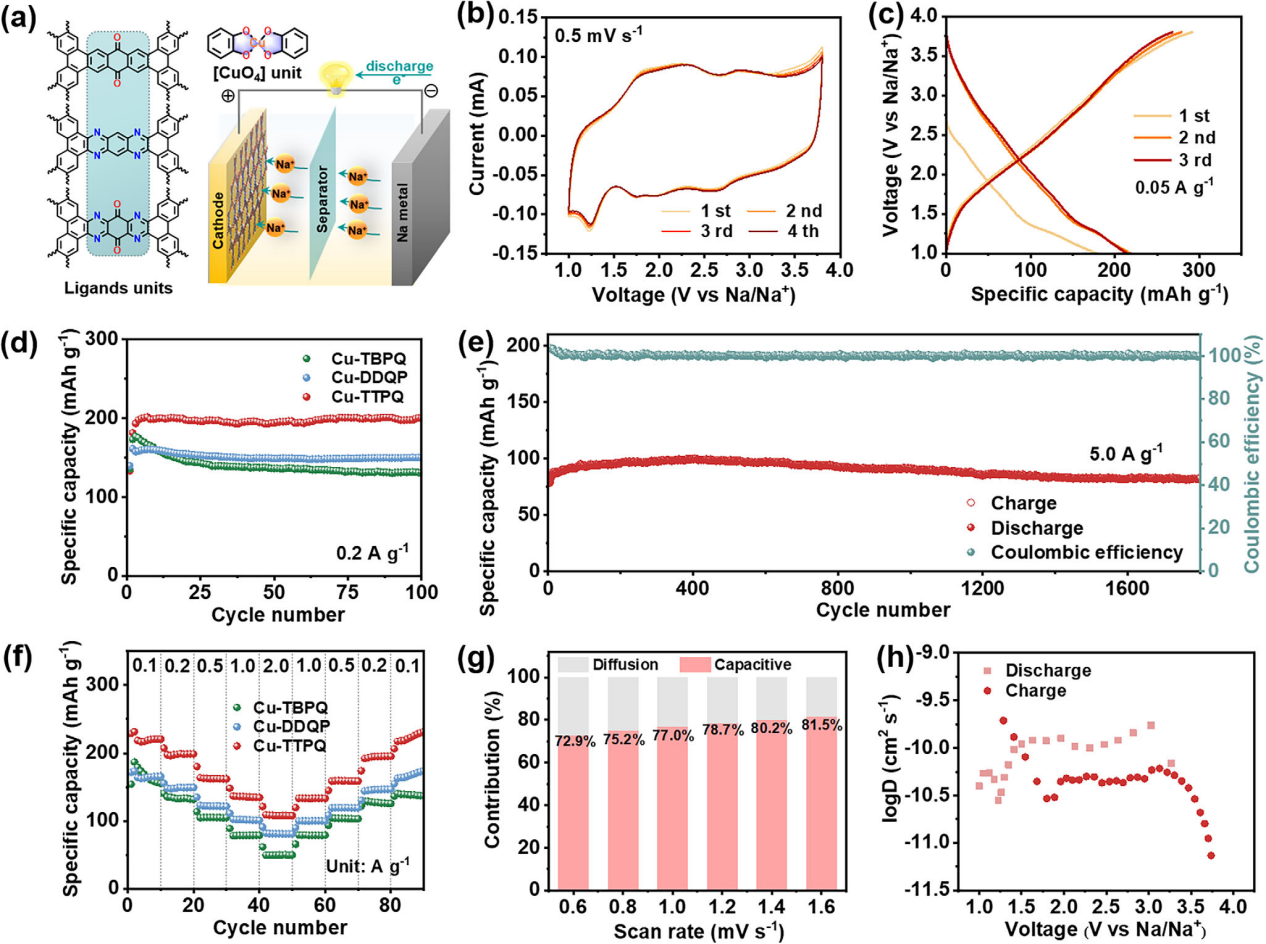
Electrochemical performance of 2D *c*‐MOFs as cathode materials. a) Schematic diagram of SIBs. b) CV curves of Cu‐TTPQ at 0.5 mV s^−1^. c) Typical GCD profiles of Cu‐TTPQ at 0.05 A g^−1^. Cycling performance of d) three 2D *c*‐MOFs at 0.2 A g^−1^ and e) Cu‐TTPQ at 5.0 A g^−1^. f) Rate capacity of Cu‐TBPQ, Cu‐DDQP and Cu‐TTPQ. g) Diffusion and capacitive currents contributed to the charge storage of Cu‐TTPQ at different rates of 0.6‐1.6 mV s^−1^. h) Calculated Na^+^ diffusion coefficients of Cu‐TTPQ.

The electrochemical performance of Cu‐TTPQ, Cu‐TBPQ, and Cu‐DDQP as cathode materials for SIBs was systematically investigated by employing metallic Na as the anode and 1 m NaPF_6_ in 1,2‐dimethoxyethane (DME) as the electrolyte (**Figure** **3**a). Cyclic voltammogram (CV) curves and galvanostatic charge/discharge (GCD) profiles were recorded within a voltage range of 1.0–3.8 V (Figure 3b,c and Figures S26 and S27, Supporting Information). Notably, the CV curves of Cu‐TTPQ exhibited four pairs of quasi‐reversible redox peaks, which were attributed to changes in the valence states of copper ions (3.25/2.65 V), semiquinone/radical states of the coordination centers (2.75/2.00 V), C═O (2.25/1.75 V), and C═N (1.80/1.25 V) groups within the TTPQ units (Figure 3b).^[^ ^2^, ^3^, ^6^ ^]^ In contrast, both Cu‐TBPQ and Cu‐DDQP displayed only a pair of redox peaks ≈1.2–2.1 V, corresponding to the C═O bond in the TBPQ units or the C═N bond in the DDQP units, respectively.^[^ ^2^, ^3^ ^]^ In addition, the electrochemical reversibility of Cu‐TTPQ was confirmed by its nearly overlapping CV curves. Consequently, Cu‐TTPQ achieved an optimal specific capacity of 214.8 mAh g^−1^ at 0.05 A g^−1^ (Figure 3c). Additionally, the cycling performance of the three cathodes was investigated at different current densities (Figure 3d,e; Figure S28, Supporting Information). Remarkably, Cu‐TTPQ exhibited a specific discharge capacity of 201.5 mAh g^−1^ after 100 cycles at 0.2 A g^−1^, surpassing the capacities of Cu‐TBPQ (133.4 mAh g^−1^) and Cu‐DDQP (151.0 mAh g^−1^) at same conditions (Figure 3d). Moreover, Cu‐TTPQ demonstrated positive long‐cycle performance, maintaining a specific discharge capacity of 142.7 mAh g^−1^ after 800 cycles at 1.0 A g^−1^ (Figure S28, Supporting Information). Even at 5.0 A g^−1^, Cu‐TTPQ retained a capacity of 82.8 mAh g^−1^ with negligible decay after 1800 cycles (Figure 3e). This fine cyclability was further confirmed by the preservation of the rod‐like morphology of the Cu‐TTPQ cathode after the charging and discharging processes (Figure S29, Supporting Information).

Furthermore, the integration of quinone and pyrazine moieties into 2D *c*‐MOFs not only improved the conductivity (Figure S23, Supporting Information) but also ensured the excellent rate capability of Cu‐TTPQ (Figure 3f and Table S4, Supporting Information). Specifically, Cu‐TTPQ exhibited reversible capacities of 216.7, 199.0, 162.1, 134.6, and 107.8 mAh g^−1^ at current densities of 0.1, 0.2, 0.5, 1.0, and 2.0 A g^−1^, respectively, outperforming the other two 2D *c*‐MOFs. Notably, when the current density was reverted to 0.1 A g^−1^, the capacity of Cu‐TTPQ recovered to 218.9 mAh g^−1^, demonstrating remarkable rate performance. This impressive performance was probably attributed to the increased interlayer spacing upon Na^+^ insertion, which facilitated more greater exposure of the active sites in SIBs.^[^ ^17^ ^]^ Moreover, the reaction kinetics of Cu‐TTPQ was performed at scan rates ranging from 0.6 to 1.6 mV s^−1^ (Figure 3g and Figure S30, Supporting Information). As shown in Figure S30a (Supporting Information), the four pairs of CV oxidation/reduction peaks for the Cu‐TTPQ cathode exhibit similar profiles, and the corresponding peak regions gradually enlarge with increasing scan rates. The charge storage behaviors revealed that the *b* values for Cu‐TTPQ range from 0.70 to 0.97, as determined by plotting peak currents (*i*) against scan rates (*v*) on a logarithmic scale (Figure S30b, Supporting Information). These findings indicate that the Cu‐TTPQ cathode operates via dual mechanisms, encompassing both pseudocapacitive and diffusive contributions. In addition, the Dunn method was employed to quantify the contribution of surface capacitance and diffusion‐controlled processes. As shown in Figure 3g and Figure S30c (Supporting Information), the capacitive contribution rate of the Cu‐TTPQ cathode was 81.5% at a scan rate of 1.6 mV s^−1^, positively correlating with increasing scan rates, thereby confirming the dominant pseudocapacitive charge‐storage characteristics of Cu‐TTPQ. The Na^+^ diffusion behavior within the batteries was evaluated using the galvanostatic intermittence titration technique (GITT). As shown in Figure 3h and Figures S31 and S32 (Supporting Information), the calculated diffusion coefficients of Na^+^ in the Cu‐TTPQ cathode during the charge/discharge process slightly exceeded those of the other two 2D *c*‐MOFs. This difference was likely attributed to the high conductivity of Cu‐TTPQ, which facilitated rapid charge transfer and effective Na^+^ diffusion during the electrochemical reactions. Overall, as cathodes for SIBs, Cu‐TTPQ exhibited a higher specific capacity and an extended cycle life, which is superior to nearly all previously reported 2D c‐MOFs in the four‐coordinated mode (Table S5).^[^ ^18^, ^19^ ^]^

**Figure 4:**
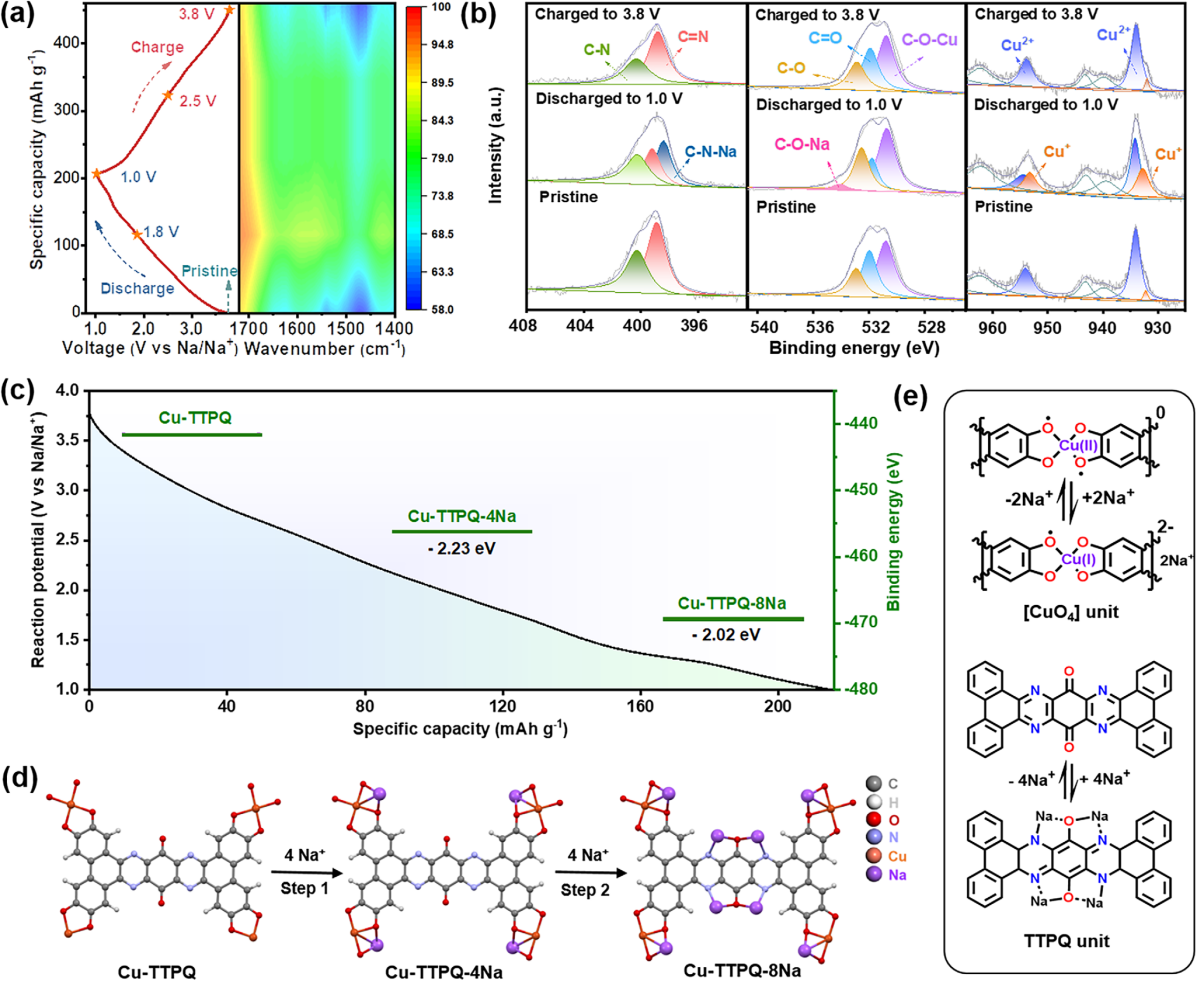
Charge storage mechanism of Cu‐TTPQ cathode. a) FT‐IR spectra. b) High‐resolution XPS spectra of N 1s, Cu 2p and O 1s at different states. c) Calculated potential pathways of Na^+^. d) Structural evolution during the insertion/extraction process of Na^+^. e) Reaction mechanism of the Na^+^ storage process in the [CuO_4_] and TTPQ units.

To investigate the Na^+^ storage mechanism of the Cu‐TTPQ cathode, ex situ FT‐IR and XPS spectra of Cu‐TTPQ during the charging/discharging processes were analyzed. As shown in **Figure** **4**a, shifts assigned to C═O/C─O and C═N/C─N bonds were observed throughout the charging and discharging processes, indicating that redox reactions occurred within the quinone and pyrazine active sites of the Cu‐TTPQ framework. Specifically, the characteristic peaks for the C═O and C═N bonds, located at ca. 1635 and 1540 cm^−1^, respectively, diminished during the discharging process, reflecting the generation of C─O/C─N and the insertion of Na^+^.^[^ ^2^, ^3^ ^]^ These changes in the C═O and C═N bonds were reversible, with Na^+^ gradually extracted from the C─O─Na and C─N─Na groups during charging, which was further corroborated by the XPS spectra. As shown in Figure 4b, the peaks associated with C═N, C═O, and Cu^2+^ at ≈398.9, 532.9, and 933.9 eV,^[^ ^2^, ^3^, ^6^ ^]^ respectively, were weakened after discharging to 1.0 V. Conversely, the corresponding peaks for C─N, C─O, and Cu^+^ at ≈400.3, 532.0, and 931.8 eV exhibited slight increases and gradually recovered upon recharging. These results indicate that C═N, C═O, and Cu^2+^ serve as active sites that simultaneously acquire electrons during the discharging process and interact with Na^+^ to form C─O─Na and C─N─Na bonds.

Further, DFT calculation was employed to simulate the multiple sodium storage processes to clarify the multi‐step Na^+^ insertion mechanism of Cu‐TTPQ cathode. Theoretically, each [CuO_4_] unit within Cu‐TTPQ have the potential to receive 2 or 3 Na^+^, denoted as Cu‐TTPQ‐4Na and Cu‐TTPQ‐6Na, respectively. Concurrently, each TTPQ unit can accommodate 4 Na^+^, denoted as TTPQ‐4Na. The optimized structures were described in Figure 4d and Figures S33 and S34 (Supporting Information), with the corresponding binding energies and calculated redox potentials of these structures listed in Table S6, Supporting Information. Notably, the binding energy of Cu‐TTPQ‐4Na (in coordination centers, −2.23 eV) is significantly more negative compared to that of Cu‐TTPQ‐4Na (in organic ligands, −2.17 eV), indicating that Na^+^ preferentially bonded with [CuO_4_] unit rather than interacting with TTPQ unit. Subsequently, multiple C═O and C═N electroactive centers cooperatively trigger the multi‐electron redox process, thereby boosting Na^+^ storage to further facilitate the specific capacity of the Cu‐TTPQ cathode. The redox potentials of Cu‐TTPQ‐6Na and Cu‐TTPQ‐10Na, corresponding to the complete transfer of three electrons in [CuO_4_], were calculated to be 0.43 and 0.07 V, respectively. Both values fell outside the selected electrochemical window, revealing that each [CuO_4_] unit accommodated 2 Na^+^. Moreover, the calculated redox potentials of Cu‐TTPQ‐4Na (2.23 V) and Cu‐TTPQ‐8Na (1.80 V) approximately aligned with the experimental discharge plateaus (2.65 and 1.75 V) presented in Figure 4c, illustrating the two‐step Na^+^ insertion mechanism within Cu‐TTPQ (Figure 4d). Consequently, Na^+^ storage during the cycling of Cu‐TTPQ was facilitated through a multi‐electron process, including two dual‐electron transfers of the [CuO_4_] units and four‐electron transfers of TTPQ multiple active sites (C═O and C═N groups) (Figure 4e). In short, per minimum unit of Cu‐TTPQ can host 8 Na^+^ owing to its multiple redox‐active sites, thereby facilitating an elevated discharge capacity. This highlights the crucial role of ligands in boosting multiple redox‐active sites, consequently enhancing energy storage.

## 3 Conclusion

In summary, we designed and synthesized three isoreticular 2D *c*‐MOFs: Cu‐TTPQ, Cu‐TBPQ, and Cu‐DDQP, which possess similar structures but differ in functional groups. The synthetic strategy for 2D *c*‐MOFs was further expanded via an in situ Scholl reaction. This approach introduces abundant C═O and C═N as redox‐active sites into the 2D *c*‐MOFs, enabling Na^+^ energy storage together with the [CuO_4_] units. Notably, as a promising cathode material for SIBs, Cu‐TTPQ exhibited a reversible capacity of 214.8 mAh g^−1^ at 0.05 A g^−1^, along with an impressive capacity retention rate of ≈99% (82.8 mAh g^−1^) after 1800 cycles at 5.0 A g^−1^. In contrast, the analogous Cu‐TBPQ and Cu‐DDQP demonstrated inferior specific capacities and cycling stability, likely because of their lower densities of active sites. In summary, the expanded one‐pot strategy for the simple synthesis of 2D *c*‐MOFs will offer convenience for large‐scale synthesis and cost control in production processes. The facile strategy for enhancing the electrochemical performance of 2D *c*‐MOF cathode materials through the integration of quinone and pyrazine structures will also pave the way for innovative designs of high‐performance 2D *c*‐MOFs for SIB applications.

## Conflict of Interest

The authors declare no conflict of interest.

## Supporting information



Supporting Information

## Data Availability

The data that support the findings of this study are available from the corresponding author upon reasonable request.
