# Supplementary material for: Two dimensional Conjugated Metal–Organic Frameworks with Multiple Redox‐Active Sites towards High‐Performance Sodium‐Ion Battery
Source: Adv Sci (Weinh). 2025 Apr 25;12(26):2503369. doi: 10.1002/advs.202503369 (PMC12245031; doi:10.1002/advs.202503369)
Supplement: Supplementary file 1 — Supporting Information [file ADVS-12-2503369-s001.docx]

Supporting Information

**2D Conjugated Metal–Organic Frameworks with Multiple Redox-Active Sites towards High-Performance Sodium-Ion Battery**

*Meiling Qi,*^#^ *Linqi Cheng,*^#^ *Xupeng Zhang, Yuzhao Guo, Xi Su, Xiaoxiao Sun, Yi Liu,* Lei Wang, Heng-Guo Wang,* and Long Chen****

**Contents**

**Section 1. Materials and Characterizations**

**Section 2. Synthetic Procedures**

**Section 3. Calculation Methods**

**Section 4. Characterization of Monomers**

**Section 5. Control Experiments of Scholl Reaction**

**Section 6. IR and UV-vis-NIR Spectra**

**Section 7. PXRD Patterns and Structural Analysis**

**Section 8. SEM and TEM Images**

**Section 9. XAS Measurements**

**Section 10. XPS Analysis**

**Section 11. Chemical Stability Tests**

**Section 12. Thermal Stability Test**

**Section 13. N_2_ Sorption Isotherms and Pore Size Distribution**

**Section 14.** **Electrical Conductivity Measurements**

**Section 15. LUMO-HOMO Energy Levels of the Cyclodehydrogenated Ligands**

**Section 16. Electrochemical Properties**

**Section 17. Reaction and Diffusion Kinetics**

**Section 18. Structural Calculations and Optimizations of Cu-TTPQ-*n*Na**

**Section 19. Supporting References**

**Section 1. Materials and Characterizations**

All purchased chemicals were analytical grade and were used without further purification, including 1,2-dimethoxybenzene, oxalyl chloride ((COCl)_2_), hydrazine hydrate, 1,2,4,5-benzenetetraamine tetrahydrochloride, chloranil, 3,4-dimethoxyphenylboronic acid, pyridine hydrochloride, 1,2,4,5-tetrabromobenzene, tetrakis(triphenylphosphine)palladium(0) (Pd(PPh_3_)_4_), potassium carbonate, iron chloride (FeCl_3_), boron tribromide (BBr_3_), copper(II) acetylacetonate (Cu(acac)_2_), cupric nitrate trihydrate (Cu(NO_3_)_2_·3H_2_O), sodium hexafluorophosphate (NaPF_6_), dimethoxyethane (DME), diethyleneglycoldimethylether (DIGLYME), ethylene carbonate (EC), diethyl carbonate (DEC), polyvinylidene fluoride (PVDF), *N*-*N*-dimethylformamide (DMF), 1,2-dichloroethane (DCE), dimethylacetamide (DMAc), anhydrous dichloromethane (DCM), ethyl acetate (EA), nitromethane, acetone, ethanol, etc.

Solution ^1^H and ^13^C NMR spectra of all monomers and intermediates were performed on a Bruker AVANCE III-400MHz NMR spectrometer at room temperature. The UV-vis-NIR spectra were measured using a Lambda 20 spectrophotometer. The Powder X-ray diffraction (PXRD) patterns for structural analysis were recorded on an X-ray diffractometer (RIGAKU SMARTLAB 9KW) with a Cu target tube and a graphite monochromator. PXRD patterns of the stability measurements were collected on a DX-27 mini 600 W X-Ray diffractometer. Fourier transform infrared (FT-IR) spectra were recorded in ATR mode on a Bruker Alpha spectrometer within the range of 400-4000 cm^−1^. The samples for transmission electron microscopy (TEM) were dispersed in ethanol. A droplet of the suspension was transferred onto a carbon-coated copper grid. High-resolution images of the 2D *c*-MOFs powders were obtained using Talos F200X and SPECTRA200 microscopes at 200 kV. X-ray photoelectron spectroscopy (XPS) was performed using a Thermo ESCALAB 250XI. Valence band (VB)-XPS was performed on a Thermo Scientific K-Alpha instrument (*Φ* = -4.2 eV). The powder pellet (3 mm × 3 mm × 1 mm) was fixed and measured at a working voltage of 12 kV, filament current of 6 mA, scanning energy of 50 eV, step size of 0.1 eV and spot diameter of 400 μm. The X-ray absorption fine structure (XAFS) spectra were collected at 4B9A beamline of Beijing synchrotron radiation facility (BSRF). The Cu K-edge (8979 eV) was measured on a bending magnet beamline. The experiments were performed in the transmission mode with argon- and N_2_-filled ionization chambers. The edge energy is associated with the maximum of the first derivative of the X-ray absorption near-edge structure (XANES) spectrum. Athena 0.9.26 was used to normalize and calibrate the data, and Artemis 0.9.26, to simulate the spectra of the model structures determined by density functional theory. The specific surface area was calculated from the nitrogen adsorption data using Brunauer-Emmett-Teller (BET) method, and the pore size distribution was calculated from the nitrogen adsorption branch using the non-local density functional theory (NLDFT) method. Elemental analysis (EA) was conducted usin a PerkinElmer 240 C elemental analyzer. Thermal gravimetric analysis (TGA) was carried out on a differential thermal analysis instrument (TA Instruments TGA Q50-1918 analyzer) over the temperature range of 20 to 800 °C under a N_2_ atmosphere with a heating rate of 10 °C min^−1^ using an empty Al_2_O_3_ crucible as the reference. Nitrogen sorption isotherms were measured using a PhysiChem iPore 620 analyzer at 77 K. The samples were activated at 80 °C for 12 h under vacuum (10^−5^ bar) prior to gas sorption analysis. The surface areas were calculated from the nitrogen adsorption data. The HOMO and LUMO energy levels of the three 2D *c*-MOFs were obtained from the valence band (VB)-XPS and UV-vis-NIR absorption spectra.

Electrical conductivity measurements were conducted using the two-probe method with a Keithley model 4200-SCS source meter. For the electrical conductivity measurements, two pieces of gold wire were attached to the side of the sample and fixed using a silver colloid that contacted the source meter. The current-voltage (*I-V*) curve measurements were performed by sweeping the voltage in the range of −1.0 to 1.0 V at room temperature.

**Section 2. Synthetic Procedures**

**2.1 Synthesis of the ligands**


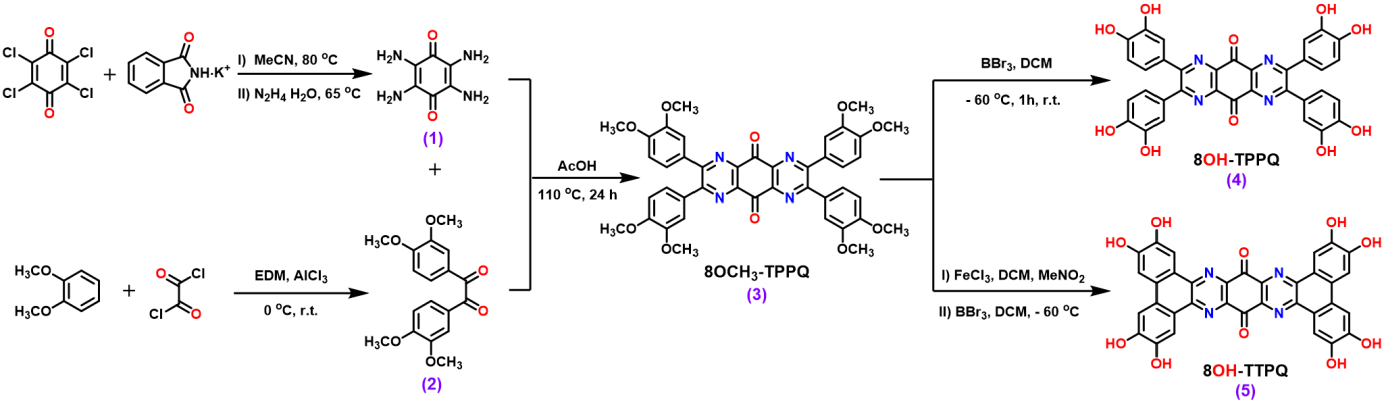


**Scheme S1.** Synthetic route of 8OH-TPPQ and 8OH-TTPQ.

**2,3,5,6-tetraaminobenzoquinone (1)** and **1,2-bis (3,4-dimethoxyphenyl) ethanedione (2)** were synthesized according to the reported literatures.^[1-2]^

**2,3,7,8-tetrakis(3,4-dimethoxyphenyl)pyrazino[2,3-g]quinoxaline-5,10-dione (8OCH_3_-TPPQ):** Under a nitrogen atmosphere, 42 mL of acetic acid was added to a 250 mL double-necked flask with compound **(1)** (700 mg, 4.2 mmol) and compound **(2)** (2.8 g, 8.5 mmol). The mixture was refluxed at 110 °C for 24 h, cooled to room temperature, poured into 500 mL of deionized water, and extracted with DCM. The organic phase was dried over anhydrous MgSO_4_, concentrated under vacuum, and purified by column chromatography using DCM: EA (6:1, *v*/*v*) as the eluent. An orange powder (1.5 g) was obtained in 47.2% yield. ^1^H NMR (400 MHz, CDCl_3_) *δ* (ppm) 7.36 (dd, *J* = 8.4, 2.0 Hz, 4H), 7.31 (d, *J* = 2.0 Hz, 4H), 6.86 (d, *J* = 8.5 Hz, 4H), 3.92 (s, 12H), 3.78 (s, 12H). ^13^C NMR (100 MHz, DMSO-*d*_6_) *δ* (ppm) 179.33, 156.35, 151.17, 148.96, 141.37, 129.65, 123.93, 112.93, 110.98.

**2,3,7,8-tetrakis(3,4-dihydroxyphenyl)pyrazino[2,3-g]quinoxaline-5,10-dione (8OH-TPPQ):** Under a nitrogen atmosphere, 8OCH_3_-TPPQ (200 mg, 0.27 mmol) and 12 g pyridine hydrochloride were placed in a 50 mL pressure tube. The mixture was refluxed at 230 ℃ for 5 h and then cooled to room temperature. The mixture was precipitated by ultrasonic dispersion in 200 mL deionized water. The solid was filtered and washed with deionized water until colorless. A red solid (130 mg) was obtained after drying under vacuum for 12 h in 75.5% yield. ^1^H NMR (400 MHz, DMSO-*d*_6_) *δ* (ppm) 9.49 (s, 4H), 9.33 (s, 4H), 7.23 (s, 4H), 6.89 (d, *J* = 7.4 Hz, 4H), 6.74 (d, *J* = 7.9 Hz, 4H). ^13^C NMR (100 MHz, DMSO-*d*_6_) *δ* (ppm) 179.51, 154.46, 147.73, 145.36, 141.43, 128.59, 121.73, 116.82, 115.52. HR-MS, m/z: Anal. Calcd. for C_34_H_20_N_4_O_10_ [M-H]^+^ 644.12, found 645.127.

**2,3,6,7,13,14,17,18-octahydroxytetrabenzo-5,7,12,14-tetraaza-6,13-pentacenequinone (8OH-TTPQ):** In a 250 mL double necked flask, the solution of anhydrous FeCl_3_ (3.0 g, 18.5 mmol) in 20 mL nitromethane was added into the solution of 8OCH_3_-TPPQ (600 mg, 0.79 mmol) in 60 mL anhydrous DCM. The mixture was then stirred for 24 h at room temperature. The crude product was deposited by adding 200 mL methanol, filtered, and washed with 100 mL methanol and 25 mL petroleum ether until colorless. The crude product was dried under vacuum for 12 h to give a red powder. Without further purification, the red powder (200 mg) was directly dissolved in 20 mL anhydrous DCM, and 2 M BBr_3_ (1.5 mL, 3 mmol) was slowly added at -20 ℃ under nitrogen. The mixture was then stirred at room temperature for 48 h. Finally, the reaction mixture was poured into ice water. The solid was collected by filtration and washed with water to obtain a red solid (140 mg) in 81% yield. ^1^H NMR (400 MHz, DMSO-*d*6) *δ* (ppm) 10.22 (s, 4H), 10.18 (s, 4H), 8.65 (s, 4H), 7.76 (s, 4H). ^13^C NMR (100 MHz, DMSO-*d*6) *δ* (ppm) 179.87, 150.95, 146.66, 142.82, 141.72, 126.92, 121.17, 110.99, 108.01. MALDI-TOF mass, m/z: Anal. Calcd. for C_34_H_16_N_4_O_10_ [M-H]^+^ 640.09, found 641.727.

**2,3,6,7-tetrakis(3,4-dihydroxyphenyl)anthracene-9,10-dione (8OH-TPAQ) and** **2,3,7,8-tetrakis(3,4-dihydroxyphenyl)pyrazino[2,3-g]quinoxaline (8OH-TPQ)** were synthesized followed the reported literature.^[3-4]^

**2.2 Synthesis of 2D *c*-MOFs**


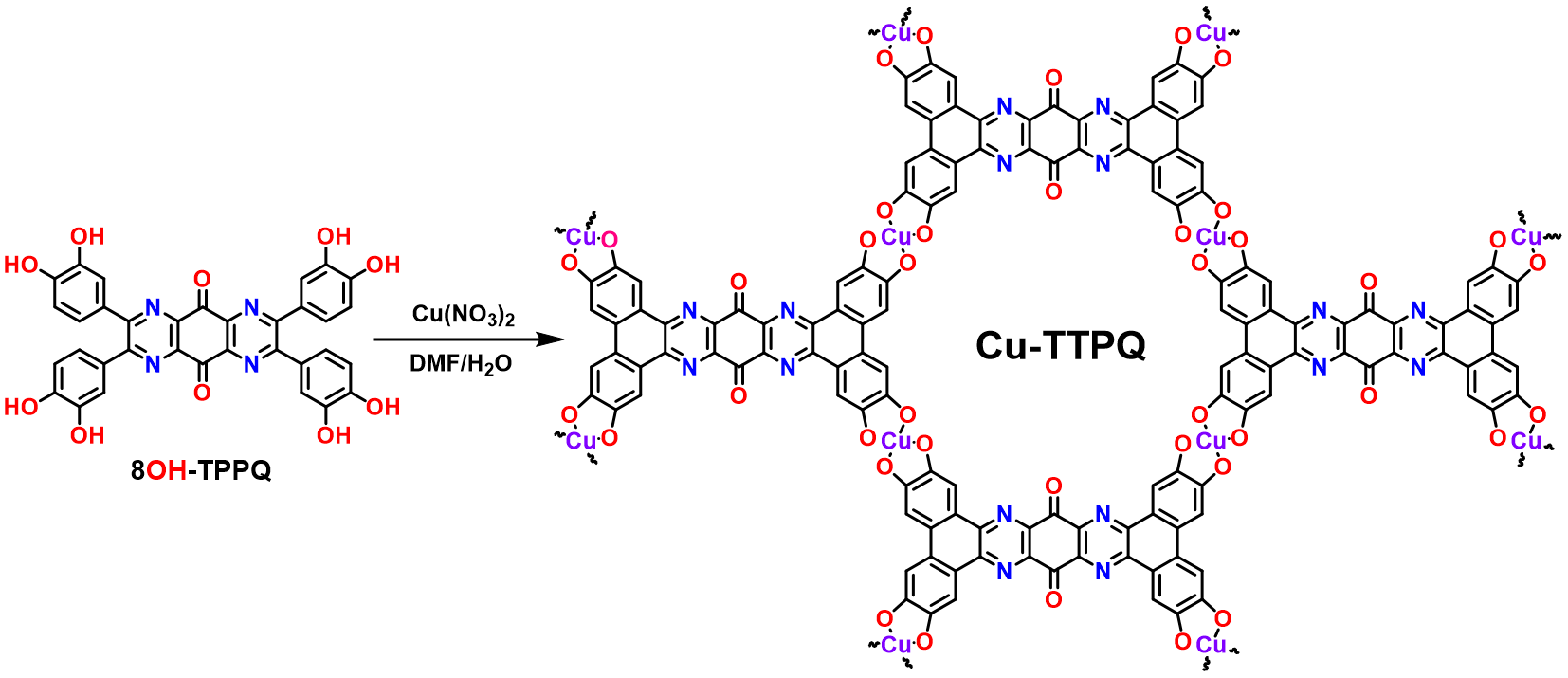


**Scheme S2.** Synthesis of Cu-TTPQ.

**Synthesis of Cu-TTPQ**

In a 10 mL glass vial, 8OH-TPPQ (13.0 mg, 0.02 mmol, 1 equiv.) and Cu(NO_3_)_2_·3H_2_O (7.3 mg, 0.03 mmol, 1.5 equiv.) were respectively dispersed in 2.0 mL DMF and 0.5 mL H_2_O by ultrasonic treatment for 15 min to obtain a black suspension. The screwed vial with the suspension was placed in a preheated oven at 85 °C for 3 days. After cooling to room temperature, the precipitate was isolated via filtration and washed with DMF, deionized water, and acetone. The solid was collected and dried under vacuum to afford Cu-TTPQ as a black powder (13.6 mg, 90%). Elemental analysis: Calcd. for Cu-TTPQ: Cu, 16.73%; C, 53.77%; H, 1.06%; N, 7.38%. Found: Cu, 16.83%; C, 48.90%; H, 3.32%; N, 9.20 %.


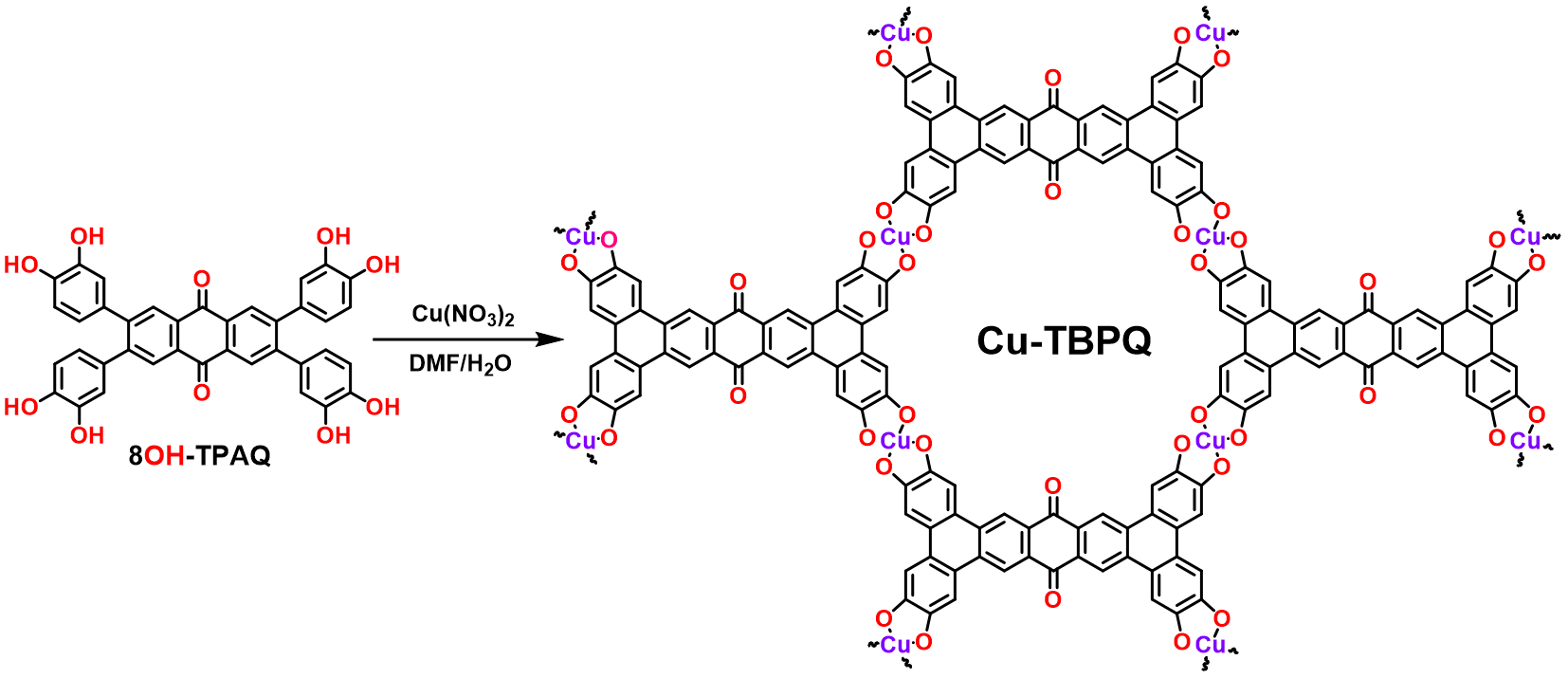


**Scheme S3**. Synthesis of Cu-TBPQ.

**Synthesis of Cu-TBPQ**

A 10 mL glass vial was charged with 8OH-TPAQ (12.8 mg, 0.02 mmol, 1 equiv.), Cu(NO_3_)_2_·3H_2_O (12 mg, 0.05 mmol, 2.5 equiv.), and DMF/H_2_O (2 mL/0.5 mL, *v*/*v* = 4:1). The vial was heated at 85 °C for three days. After cooling to room temperature, the precipitate was isolated via centrifugation and thoroughly washed with DMF, deionized water, and acetone, respectively. The solid was collected and dried under vacuum to obtain Cu-TBPQ as a black powder (14.6 mg, 96%).


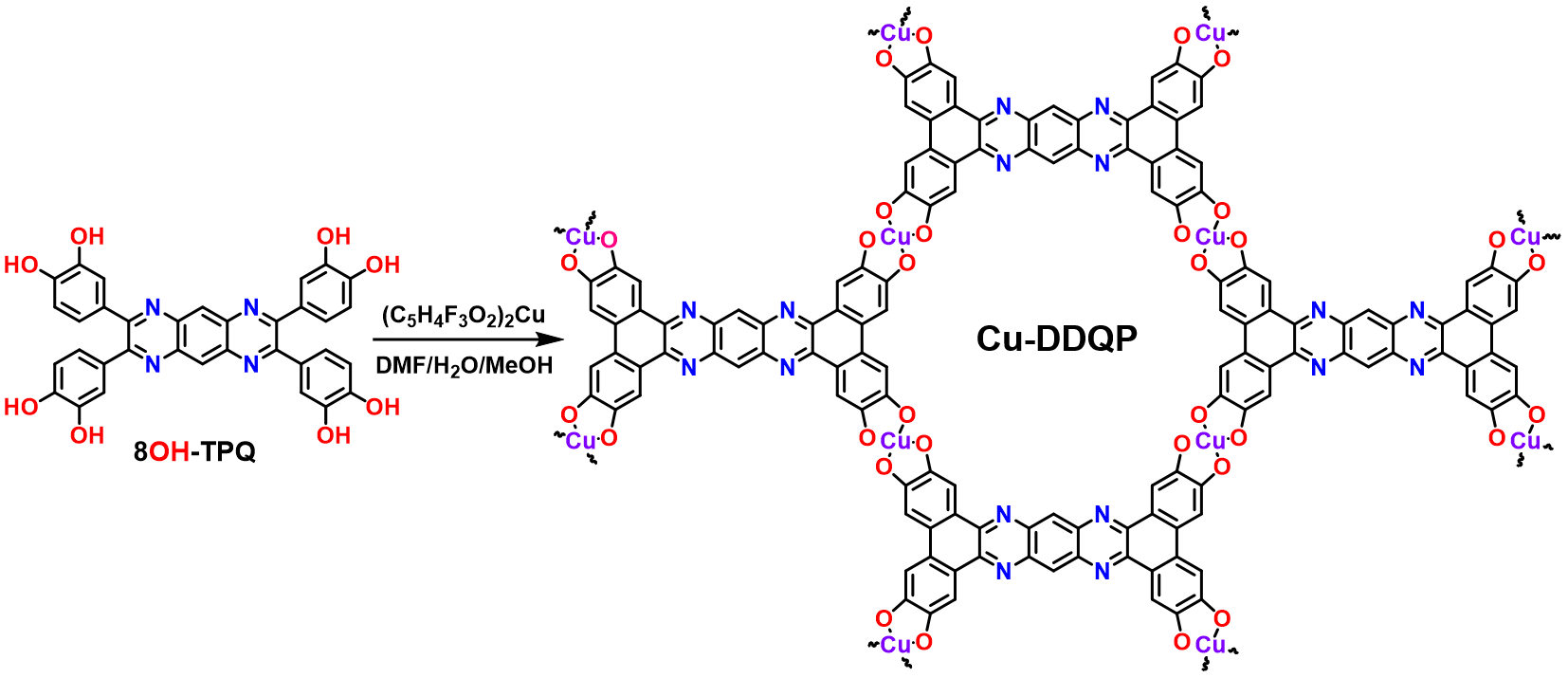


**Scheme S4**. Synthesis of Cu-DDQP.

**Synthesis of Cu-DDQP**

In a 10 mL glass vial, 8OH-TPQ (6.1 mg, 0.01 mmol, 1 equiv.) and (C_5_H_4_F_3_O_2_ )_2_Cu (7.4 mg, 0.02 mmol, 2 equiv.) were dissolved in the mixture of DMF/H_2_O/MeOH (1.0 mL/1.5 mL/0.5 mL, 2:3:1). After sonication for 30 min, the vial was placed in an oven at 85 °C for two days. After cooling to room temperature, the precipitate was isolated via filtration and washed with DMF, deionized water, and acetone, respectively. The solid was collected and dried under vacuum to obtain Cu-DDQP as a black powder (6.4 mg, 91%).

**Digestion of 2D *c*-MOFs**

The as-synthesized 2D *c*-MOF (Cu-TBPQ, Cu-DDQP or Cu-TTPQ) (10 mg) were separately dissolved in a mixture of *N,N*-dimethylformamide and 36% HCl (200 μL/40 μL, *v*/*v =* 5:1) and sonicated for 10 min. The obtained dark red solution was filtered and followed by addition of 20 mL deionized water was added. After 30 min, the resulting precipitate was collected by filtration, washed, and dried under vacuum at 100 °C overnight to obtain a red-brownish powder.

**Section 3. Calculation Methods**

**Conductivity Calculation**


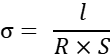


where *σ* is the conductivity, *R* is the resistance of the material, *l* and *S* are the thickness and cross-sectional area of the tested sample.

**Electrochemical Measurements**

The Cu-TBPQ, Cu-DDQP and Cu-TTPQ cathodes were prepared on aluminum foils, wherein the ratio of electrode materials, acetylene black and PVDF is around 6:3:1. Consequently, the ratio of active materials was approximately 60%, and the capacity was calculated based on the mass of the active materials. The loading amount of active materials is around 0.5 mg cm^−2^. The coin-type cells were assembled in an Ar-filled glove box with water and oxygen level less than1.0 ppm. The separator was fiber glass (Whatman GF/A) and the counter electrode was metallic Na. The electrolyte was 1 M NaPF_6_ in dimethoxyethane (DME). The galvanostatic charge/discharge (GCD) and cyclic voltammetry (CV) of the three 2D *c*-MOF-based cathodes were tested at room temperature using CR2025 coin-type batteries. CV measurements were performed using a CHI600E battery testing system (CH Instruments, Chenhua Co., Shanghai, China) with a voltage range of 1.0~3.8 V. The electrochemical measurements were evaluated by a NEWARE battery system. The chemical diffusion coefficient of Na^+^ was tested using the galvanostatic intermittent titration technique (GITT) at a current density of 0.05 A g^−1^ for 10 min followed by 30 min of relaxation.


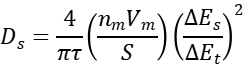


where *τ* is a relaxation time; *n_m_* is the number of moles, and the *V_m_* is the molar volume of the electrode material, and S is an electrode contact area, and Δ*E_s_* is a voltage change caused by pulses, Δ*E_t_* is the voltage change of constant current charge and discharge.

**Energy Storage Mechanism**

To determine the reaction kinetics and the charge storage mechanism of Cu-TTPQ cathodes, CV curves were tested at different scan rates from 0.6 to 1.6 mV s^−1^. Currents depending on the scan rates study obeyed the following equation:


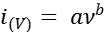


where *i*_(V)_ is the responsive current at a fixed voltage (V), *ν* is the scan rate (mV s^−1^), *a* and *b* are arbitrary coefficients. When *b* = 0.5, it represented a diffusion-controlled reaction. When *b* = 1, it was a surface capacitive reaction.

Furthermore, the respective contribution of two separate mechanisms at fixed voltage is calculated by the following equations:


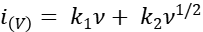


where the *k*_1_ and *k*_2_ are appropriate values, contributions of current responds from the surface capacitive reaction and diffusion-controlled reaction are expressed by *k*_1_*ν* and *k*_2_*ν*^1/2^, respectively. Dividing *ν*^0.5^ on both sides of the above equation yields:


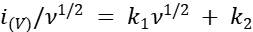


**Theoretical capacity of 2D *c*-MOFs**

In theory, the structural units of Cu-TBPQ, Cu-DDQP, and Cu-TTPQ can facilitate the transfer of 6, 8, and 8 electrons, respectively during the discharging and charging processes. Additionally, their molar weights are measured at 636.6, 610.5 and 640.5 g mol^-1^. The theoretical capacity was calculated according to the following formula:


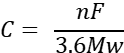


where *n* is the electron transfer number, *F* is the Faraday constant (96485 C mol^-1^), *Mw* is the Molar weight (g mol^-1^). Hence, the theoretical capacity of Cu-TBPQ, Cu-DDQP and Cu-TTPQ are measured to be 252.6, 351.2 and 334.8 mAh g^-1^, respectively.

**Section 4.** **Characterization of Monomers**

**
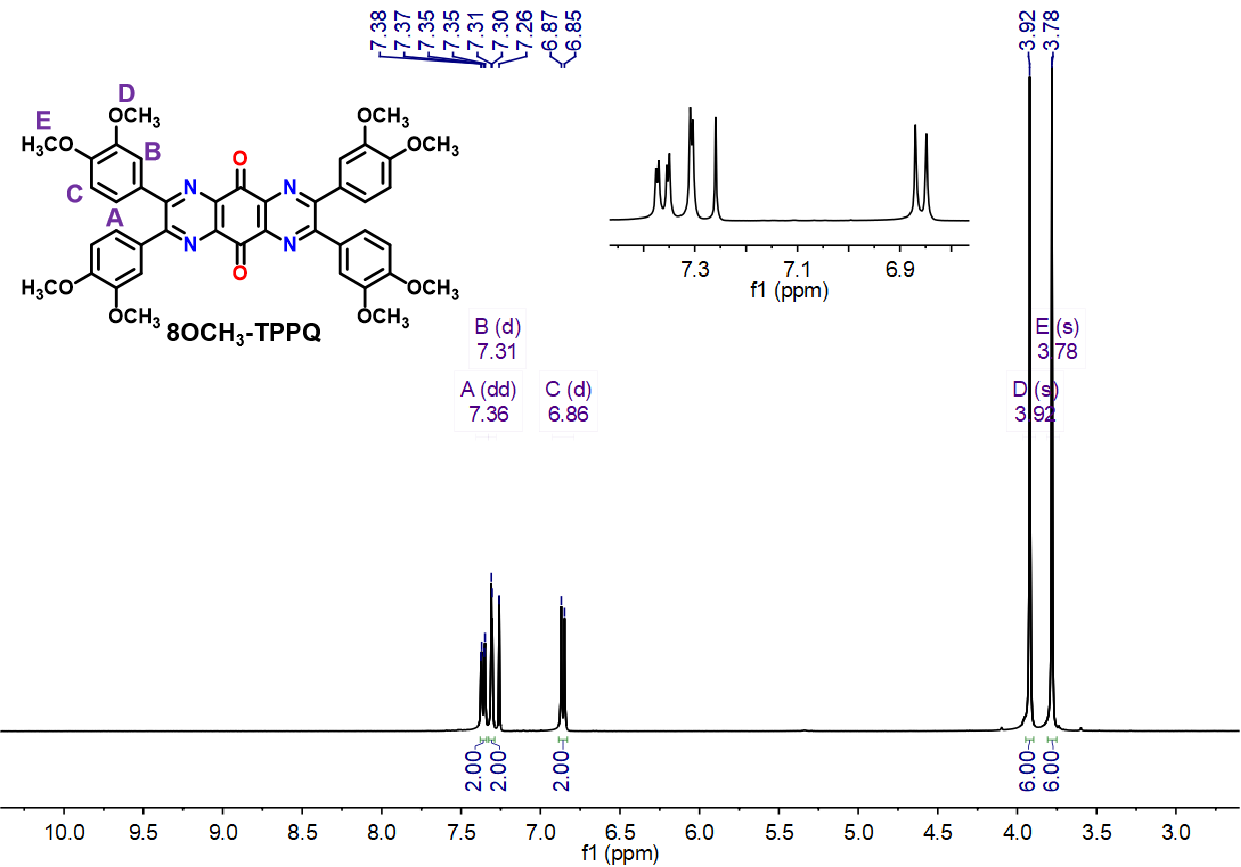
**

**Figure S1.** ^1^H NMR spectrum (400 MHz) of 8OCH_3_-TPPQ in CDCl_3_ at room temperature.

**
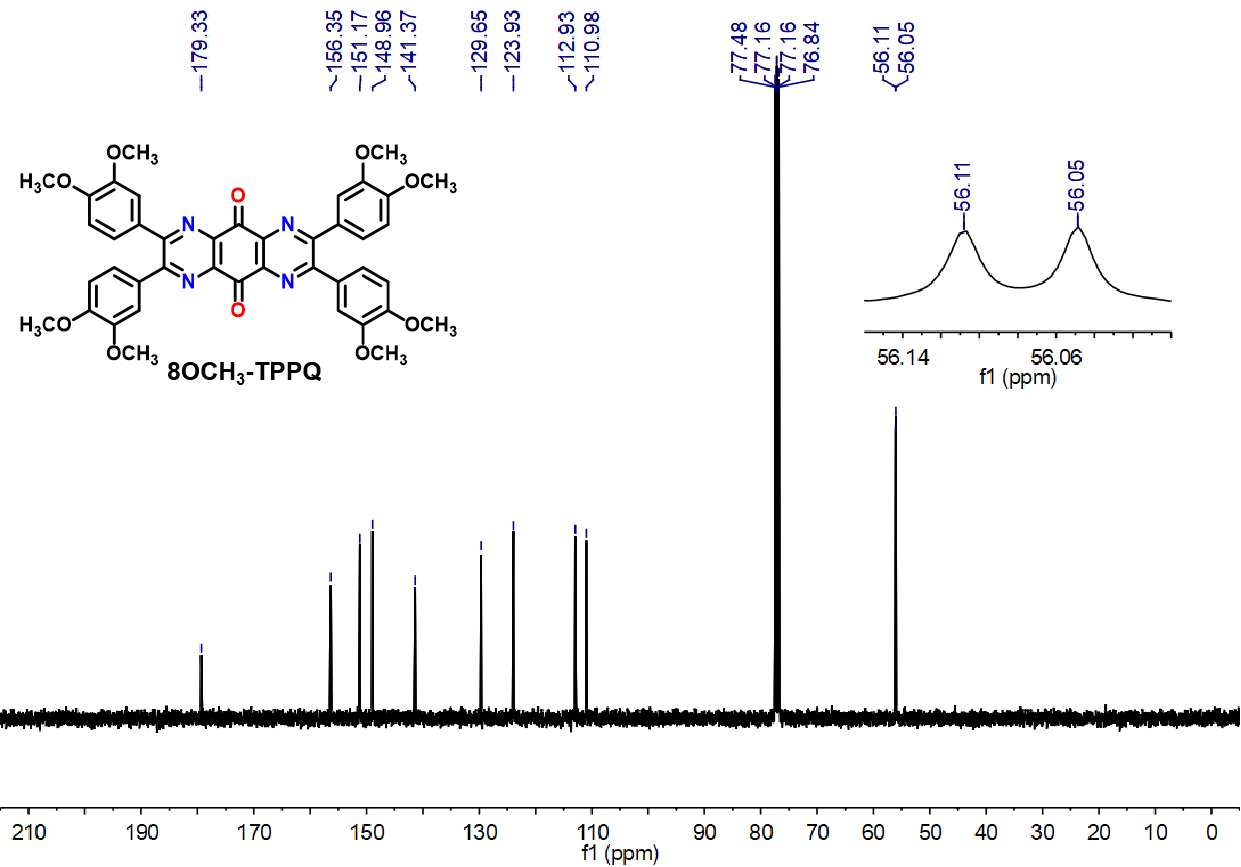
**

**Figure S2.** ^13^C NMR spectrum (100 MHz) of 8OCH_3_-TPPQ in CDCl_3_ at room temperature.

**
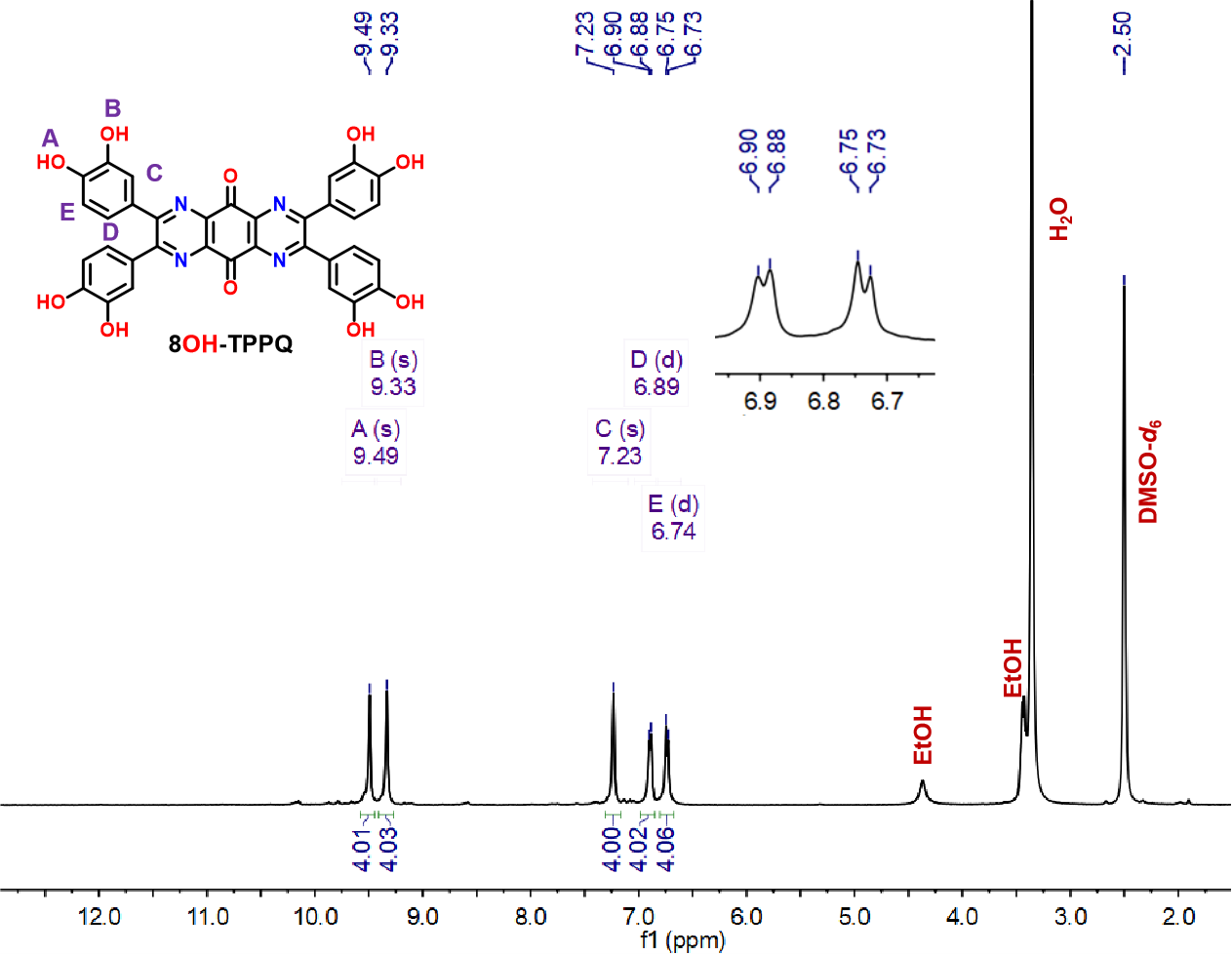
**

**Figure S3.** ^1^H NMR spectrum (400 MHz) of 8OH-TPPQ in DMSO-*d*_6_ at room temperature.

**
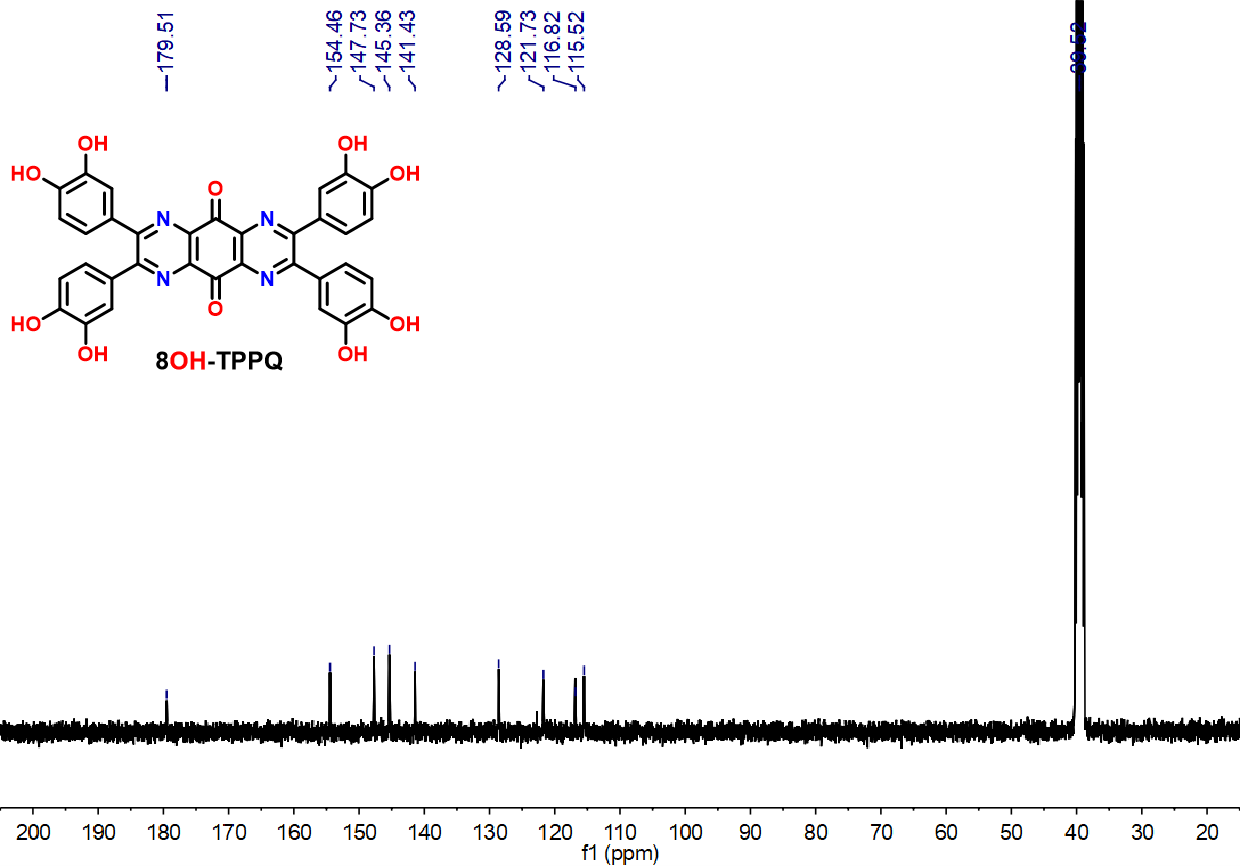
**

**Figure S4.** ^13^C NMR spectrum (100 MHz) of 8OH-TPPQ in DMSO-*d*_6_ at room temperature.

**
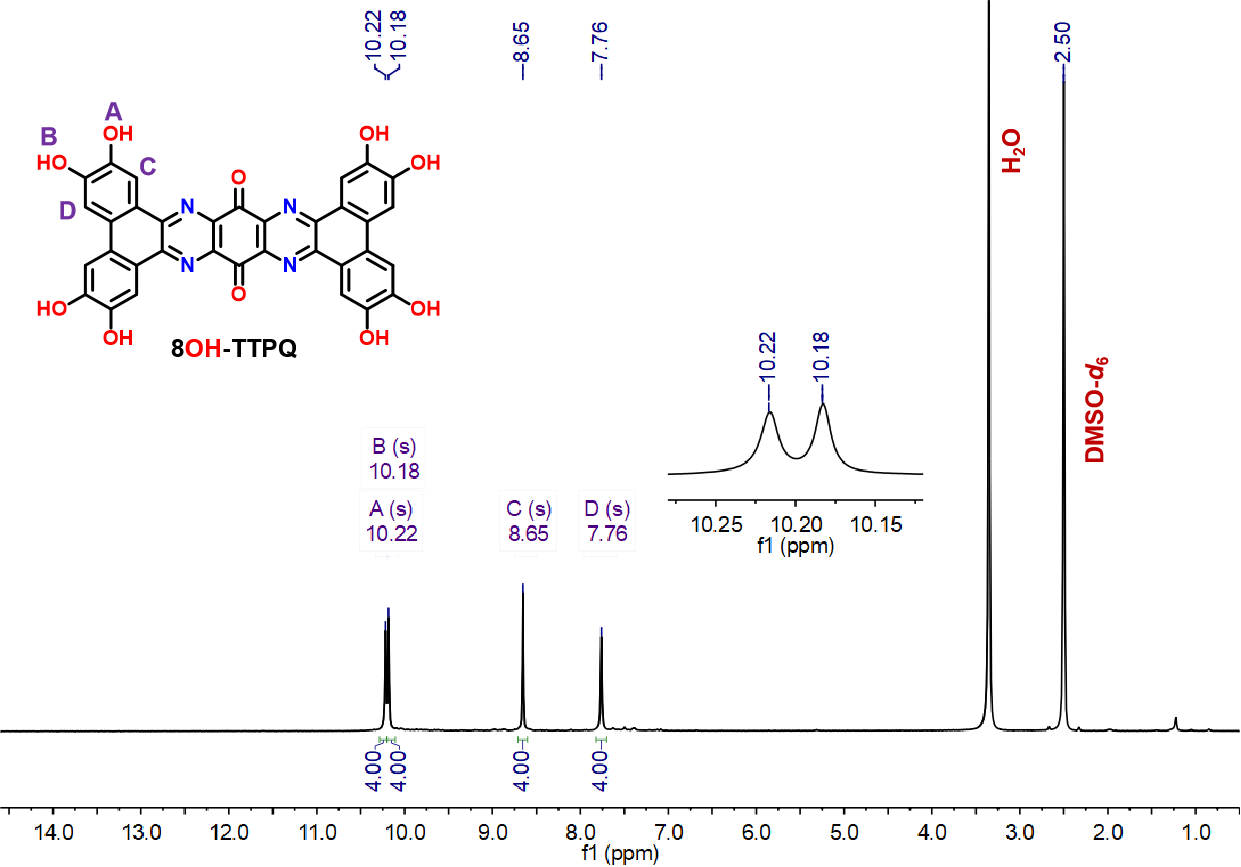
**

**Figure S5.** ^1^H NMR spectrum (400 MHz) of 8OH-TTPQ in DMSO-*d*_6_ at room temperature.

**
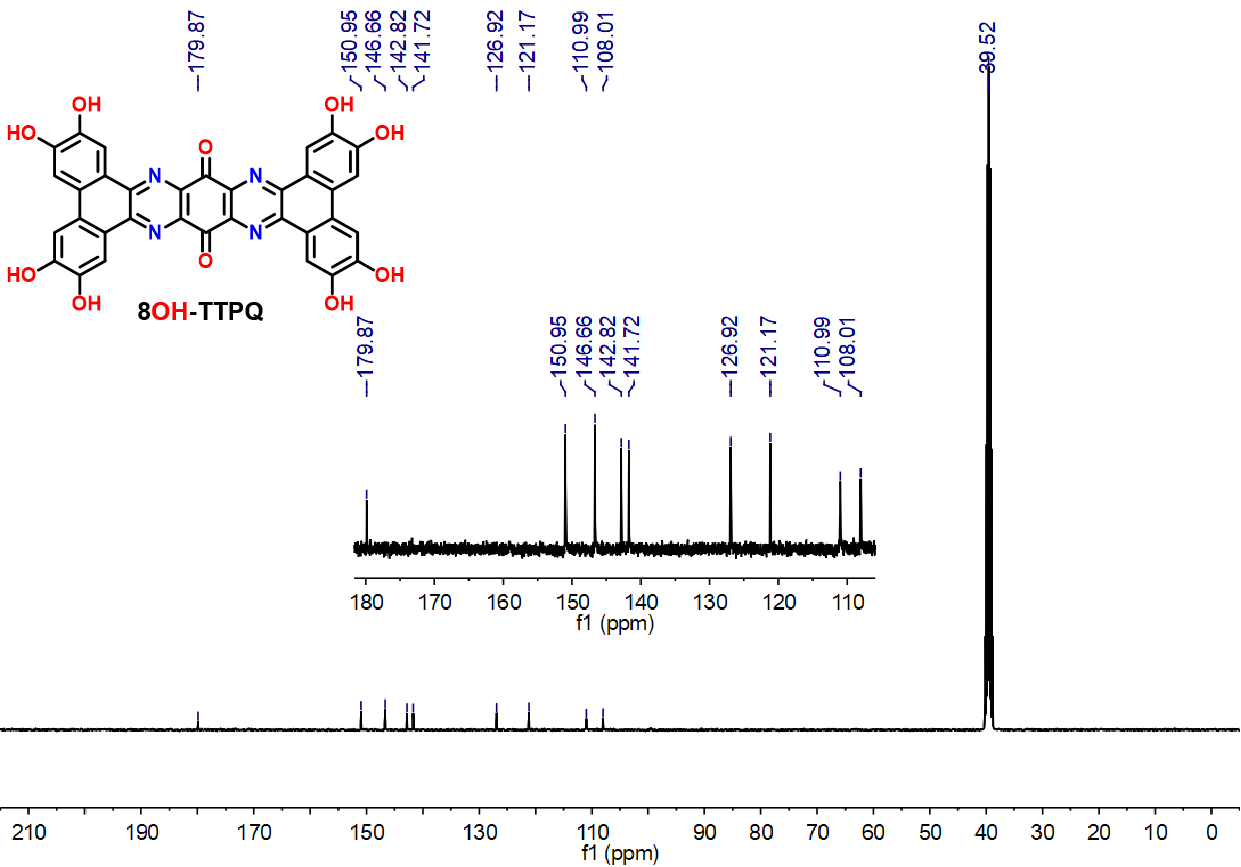
**

**Figure S6.** ^13^C NMR spectrum (100 MHz) of 8OH-TTPQ in DMSO-*d*_6_ at room temperature.

**
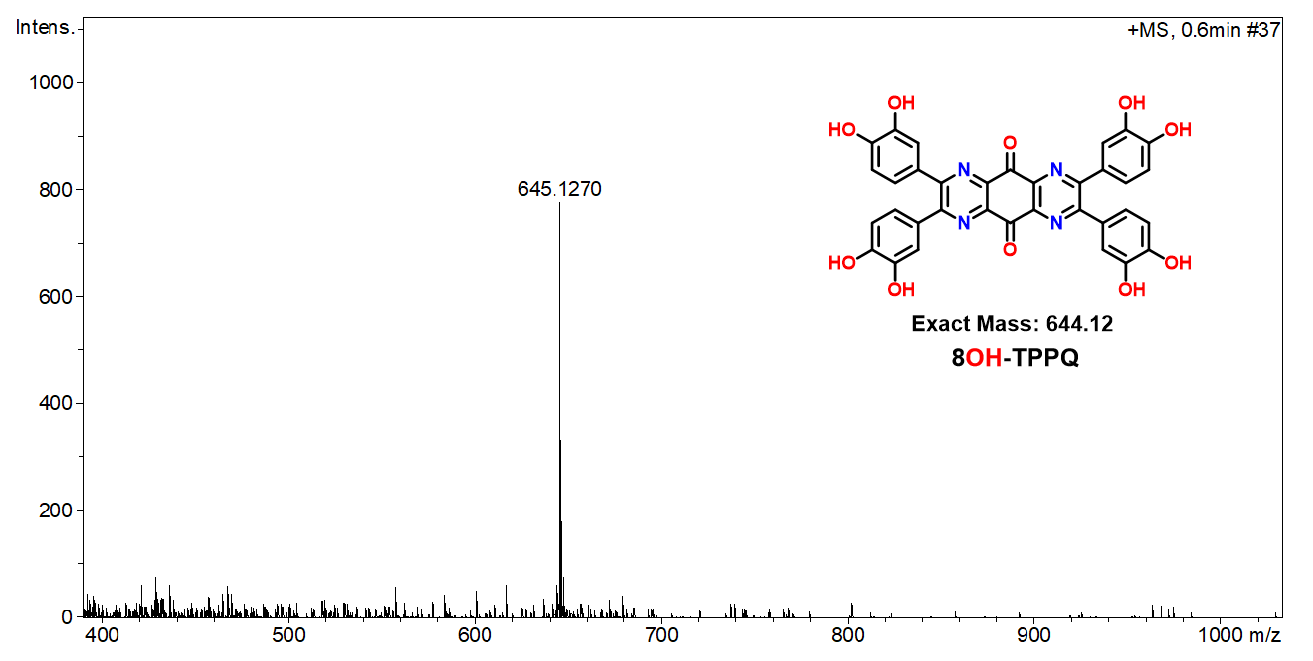
**

**Figure S7.** HR-MS spectrum of 8OH-TPPQ in MeOH.


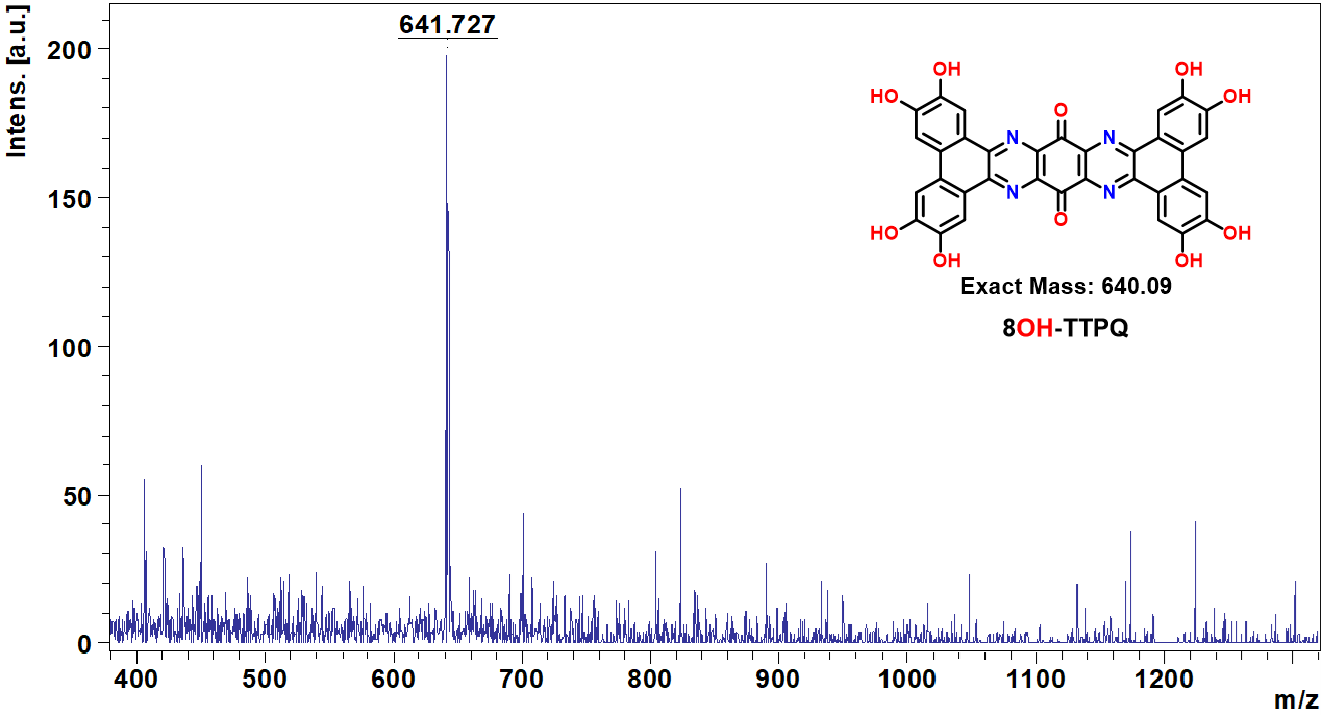


**Figure S8.** MALDI-TOF spectrum of 8OH-TTPQ in MeOH.

**Section 5. Control Experiments of Scholl Reaction**


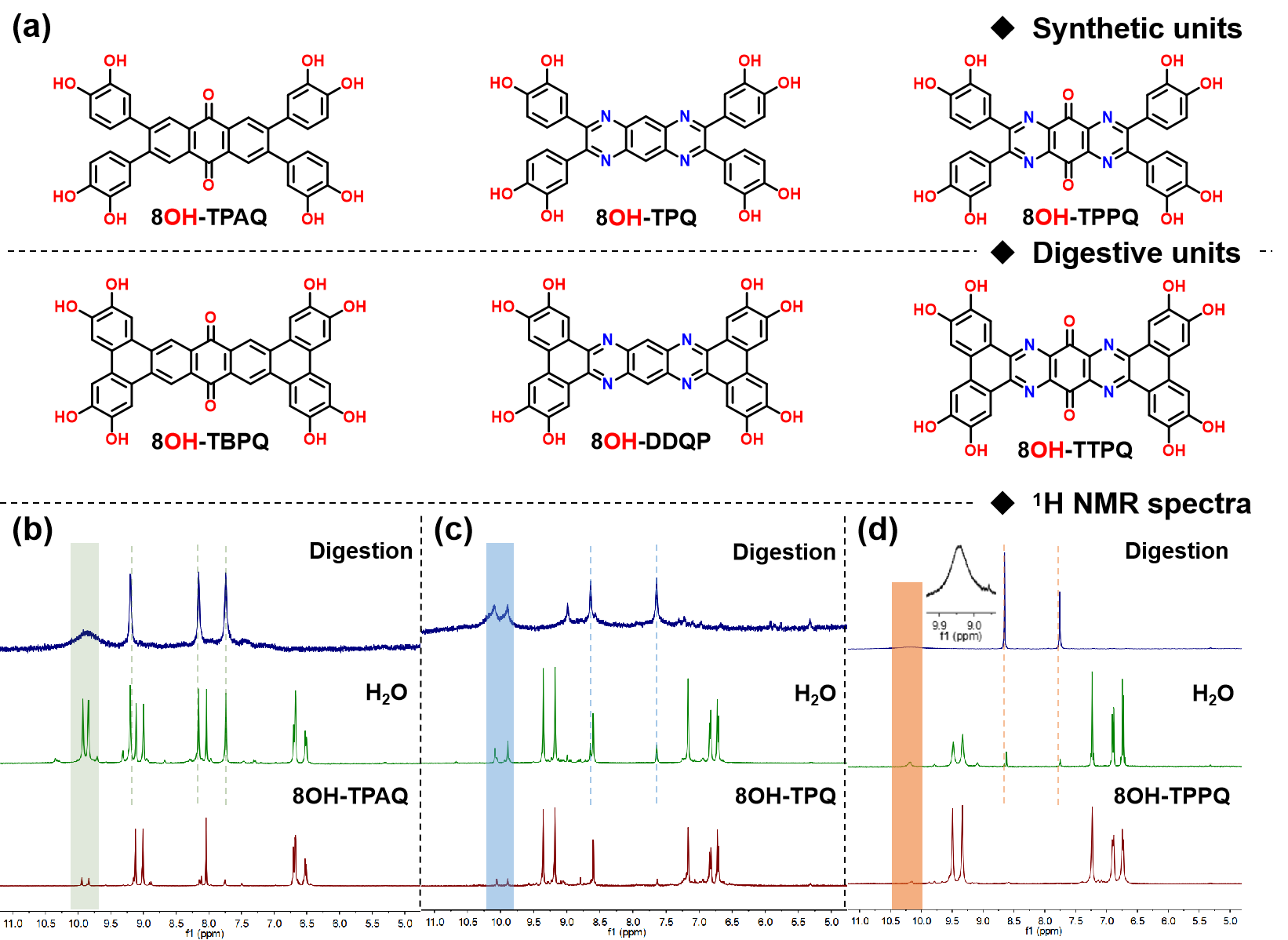


**Figure S9.** (a) Chemical structures of 8OH-TPAQ, 8OH-TPQ and 8OH-TPPQ and their respective cyclodehydrogenated counterparts of 8OH-TBPQ, 8OH-DDQP and 8OH-TTPQ. (b-d) Comparison of ^1^H NMR spectra (400 MHz) for initial 8OH-TPAQ, 8OH-TPQ and 8OH-TPPQ ligands (red lines) and their products after hydrothermal reaction^a^ (green lines) and digestion^b^ from Cu-TBPQ, Cu-DDQP and Cu-TTPQ (dark blue lines) in DMSO-*d*_6_ at room temperature.

**^a^Hydrothermal Reaction:** In a tightly sealed 10 mL vial, the 8OH-TPAQ (12.8 mg, 0.02 mmol), 8OH-TPQ (12.2 mg, 0.02 mmol) or 8OH-TPPQ (13.0 mg, 0.02 mmol) ligand was sonicated for 30 min in 2 mL deionized water to give a suspension. The above suspension was then transferred to a preheated 85 °C oven, and kept at 85 °C for 3 days.

**^b^Digestion:** Referring to the digestion of 2D *c*-MOFs for details in page S8.

**Section 6. IR and UV-vis-NIR Spectra**


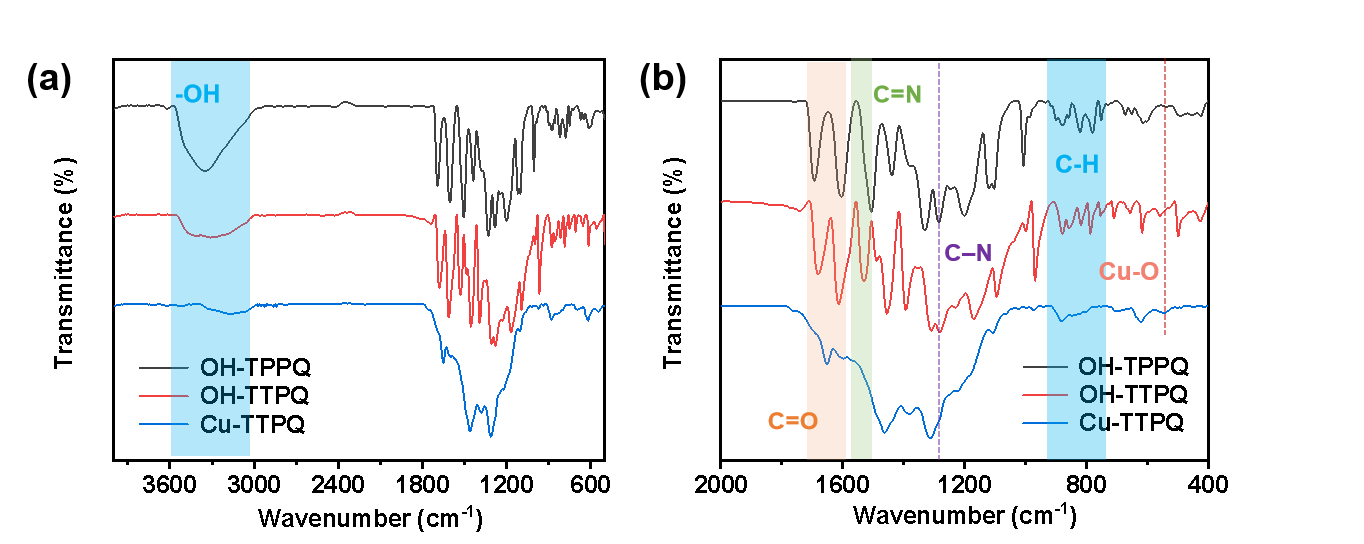


**Figure S10.** IR spectra of 8OH-TPPQ (black lines), 8OH-TTPQ (red lines) and Cu-TTPQ (blue lines) from (a) 400-4000 cm^−1^ and (b) 400-2000 cm^−1^.


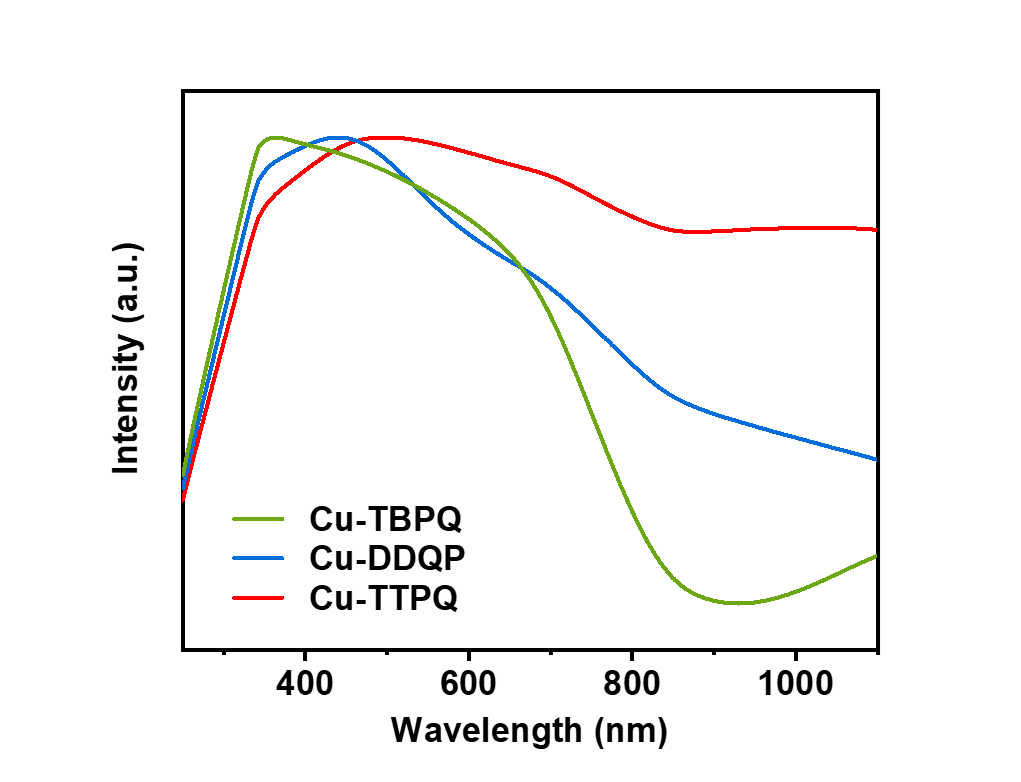


**Figure S11.** UV-vis-NIR spectra of Cu-TBPQ (green line), Cu-DDQP (blue line) and Cu-TTPQ (red line).

**Section 7. PXRD Patterns and Structural Analysis**


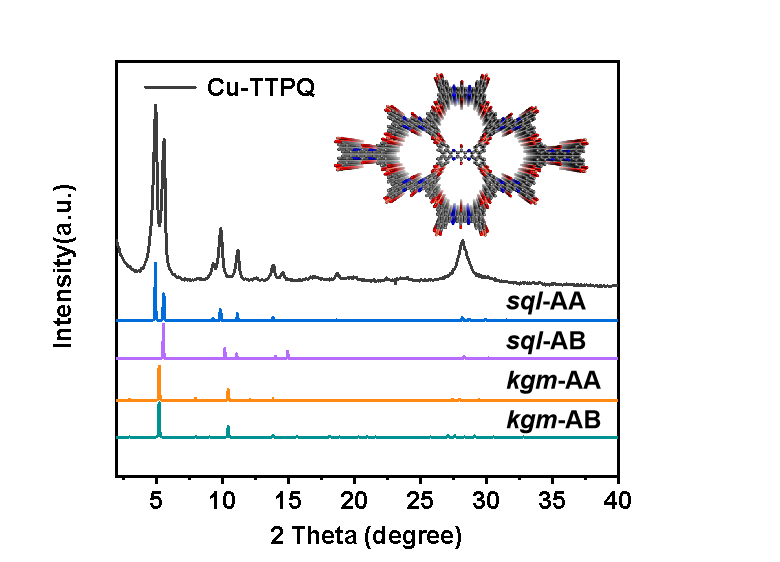


**Figure S12.** (a) Experimental (black line) PXRD and simulated PXRD patterns of *sql*-AA stacking (blue line) *sql*-AB stacking (purple line), *kgm*-AA stacking (yellow line) and *kgm*-AB stacking (green line) for Cu-TTPQ.


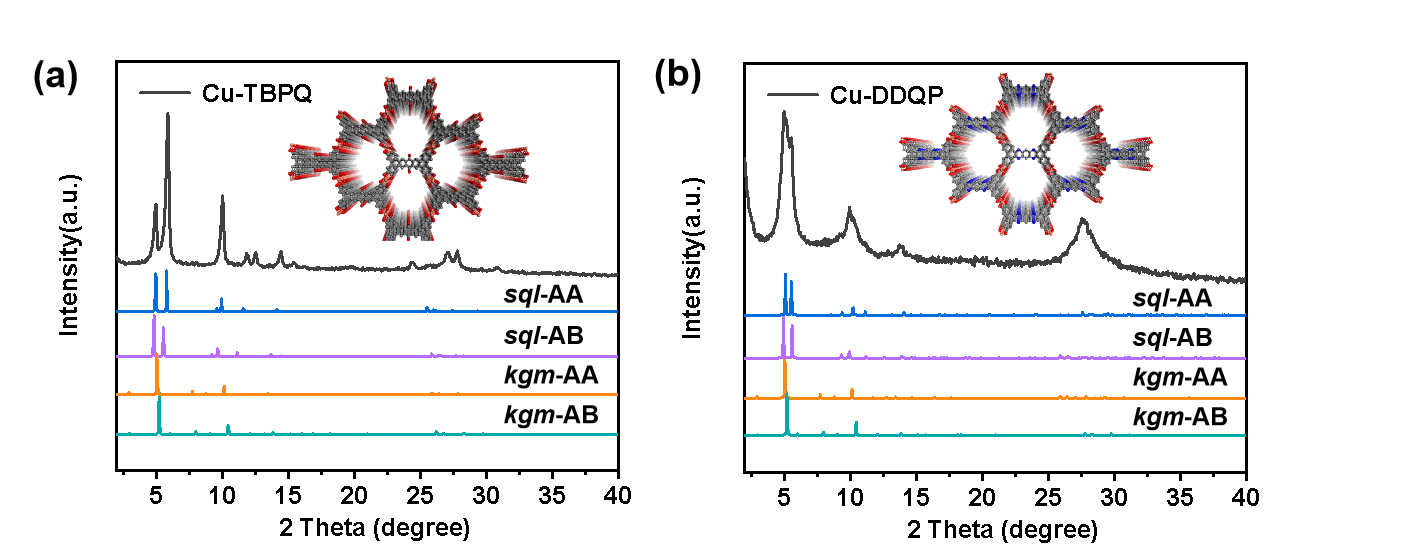


**Figure S13.** Experimental PXRD and simulated PXRD of *sql*-AA (blue lines), *sql*-AB (purple lines), *kgm*-AA (yellow lines) and *kgm*-AB (green lines) patterns for (a) Cu-TBPQ and (b) Cu-DDQP.

**Section 8.** **SEM and TEM Images**


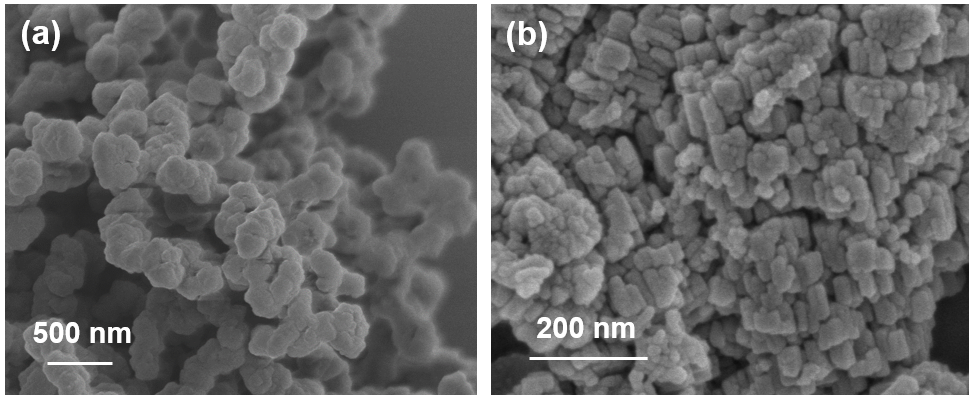


**Figure S14.** SEM images of (a) Cu-TBPQ and (b) Cu-DDQP.


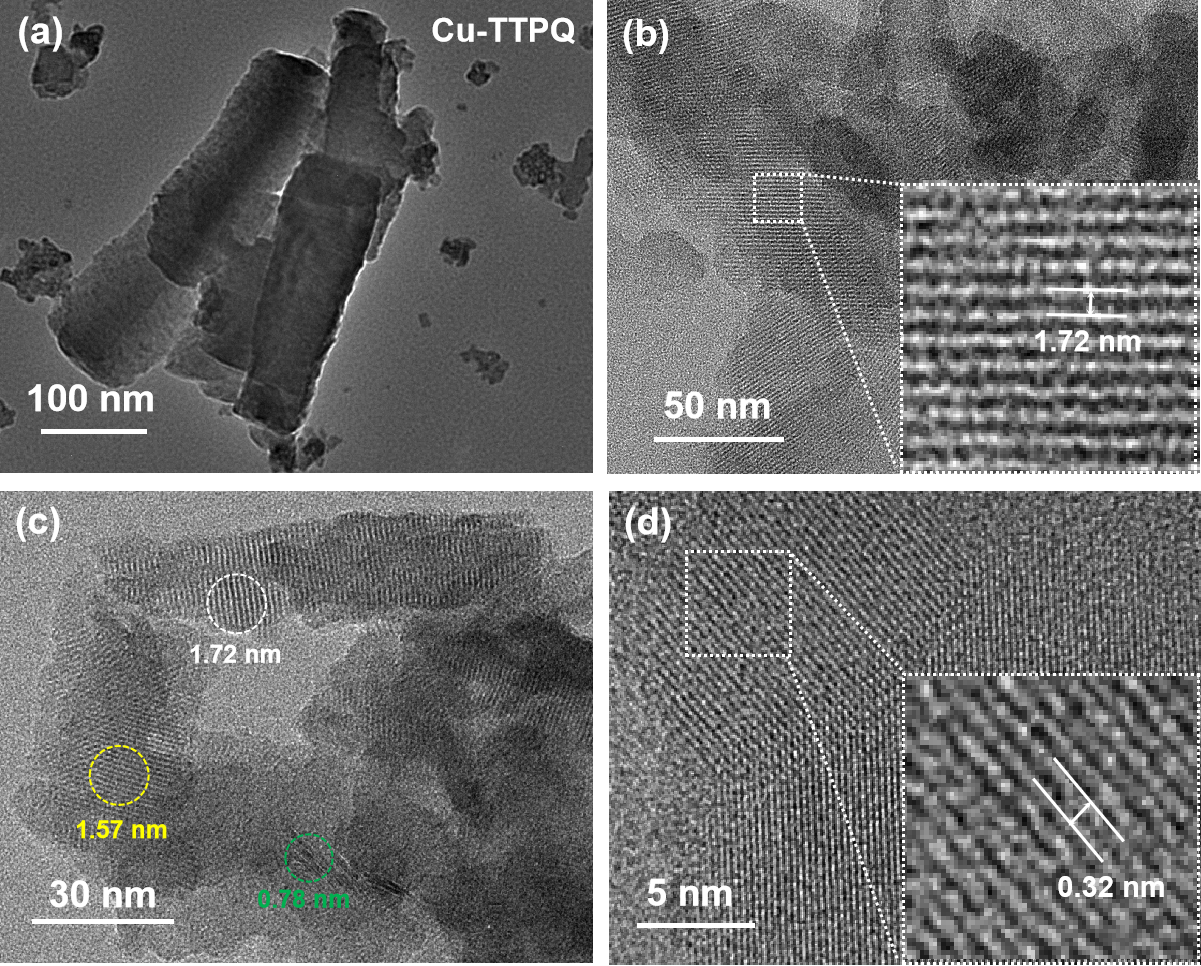


**Figure S15.** TEM images of Cu-TTPQ. a) Morphology image under lower magnification. b), c) and d) High-magnification and zoom-in view images from the white squares. The discernible lattice spacing in white, yellow and green circles correspond respectively to the *d*-spacing (17.85, 15.73, 7.91 and 3.16 Å) of the (110), (020), (040) and (001) planes.

**Section 9. XAS Measurements**


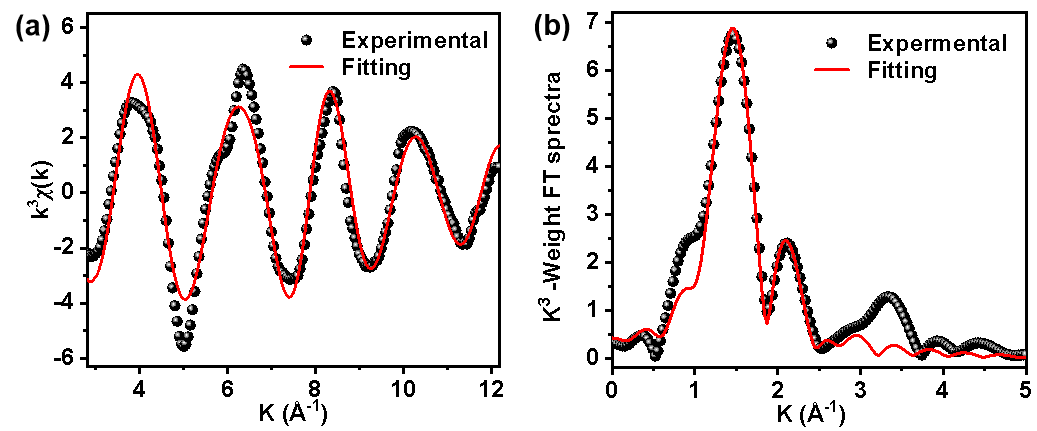


**Figure S16.** (a) Cu K-edge EXAFS and (b) FT spectrum oscillation k^3^*χ* (k) for Cu-TTPQ.

**Table S1.** Cu K-edge EXAFS fitting parameters of Cu-TTPQ.

| **Sample** | **Bond** | **R (Å)** | **N** | **σ^2^ (10^-3^ Å^2^)** | **∆E (eV)** | **R factor** |
| --- | --- | --- | --- | --- | --- | --- |
| **Cu-TTPQ** | Cu-O | 1.90 | 4.0 | 4 | -5.9 | 0.02 |
|  | Cu...C | 2.66 | 4.0 | 9 | -5.9 |  |

**Section 10. XPS Analysis**


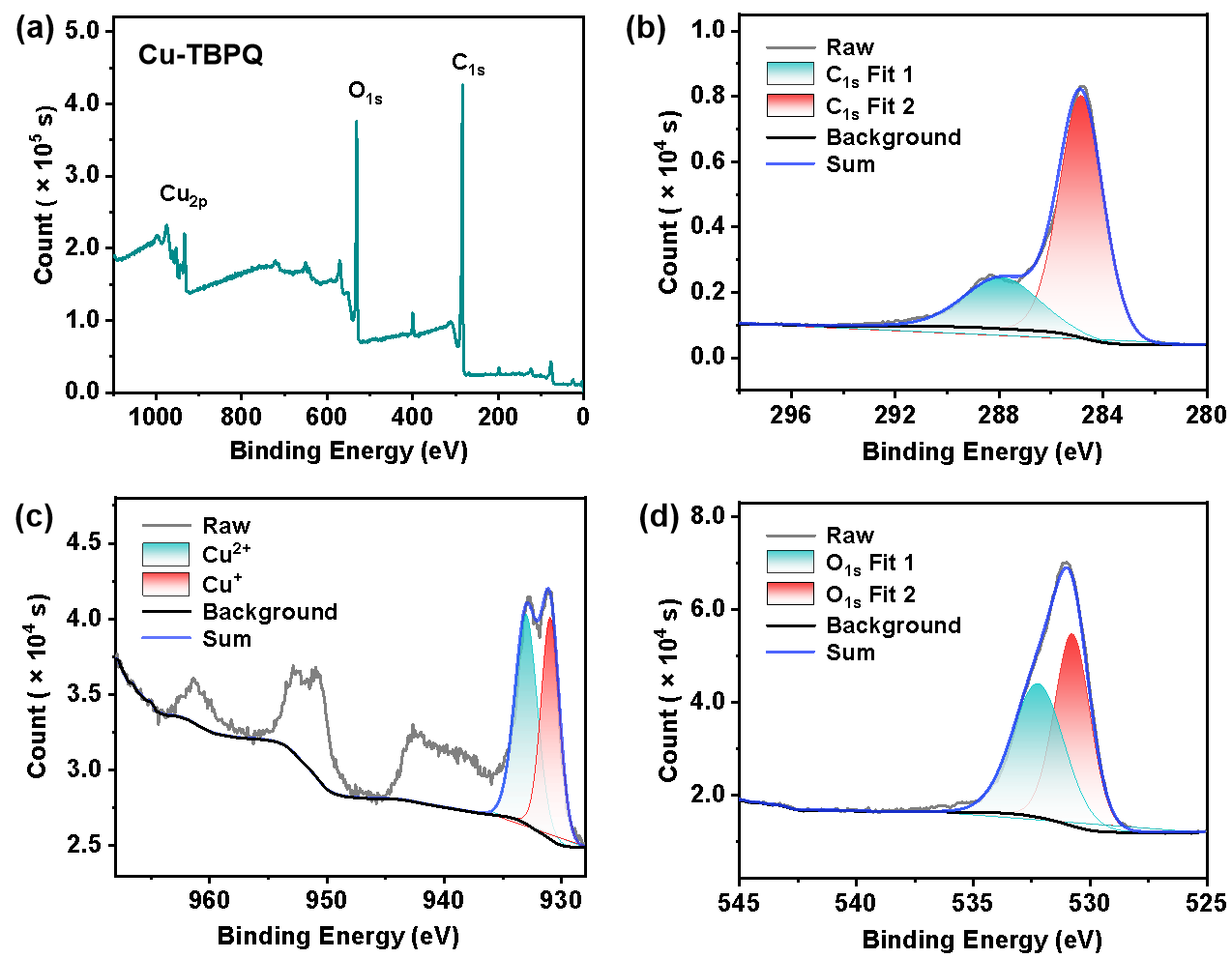


**Figure S17.** (a) XPS analysis of full energy spectrum for all the elements and high-resolution XPS spectra of (b) C(1s), (c) Cu(2p) and (d) O(1s) for Cu-TBPQ.


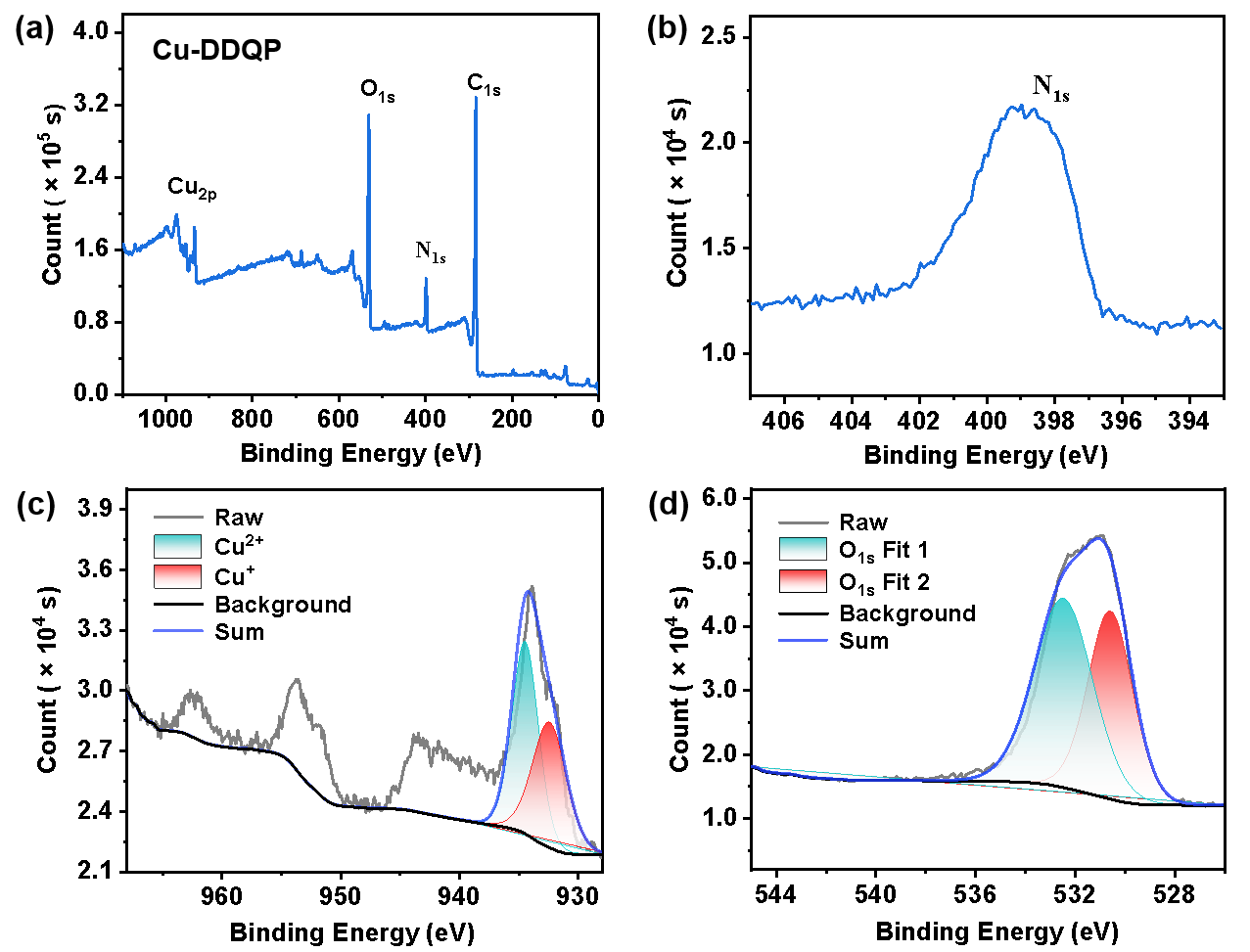


**Figure S18.** (a) XPS analysis of full energy spectrum for all the elements and high-resolution XPS spectra of (b) N(1s), (c) Cu(2p) and (d) O(1s) for Cu-DDQP.


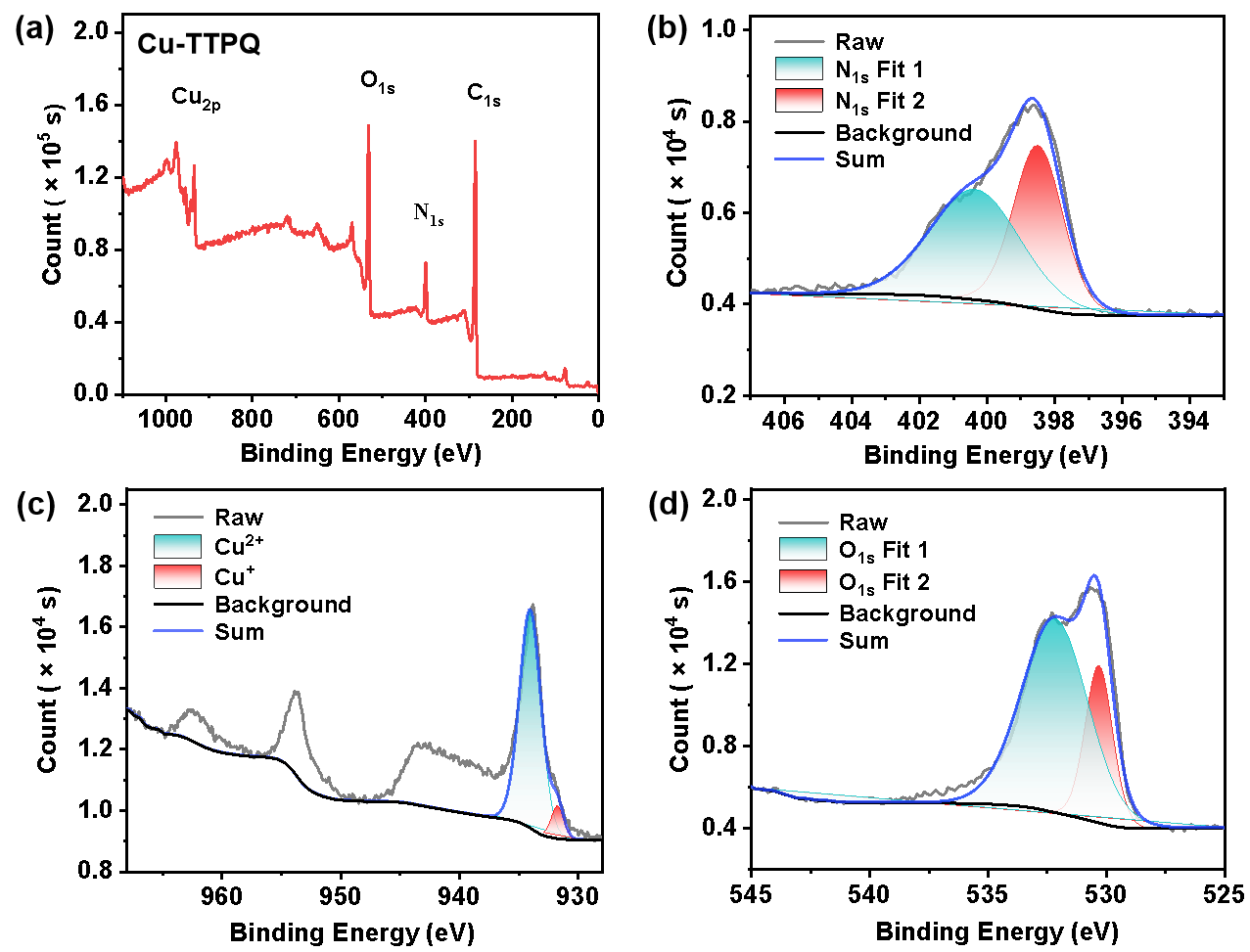


**Figure S19.** (a) XPS analysis of full energy spectrum for all the elements and high-resolution XPS spectra of (b) N(1s), (c) Cu(2p) and (d) O(1s) for Cu-TTPQ.

**Section 11. Chemical Stability Tests**


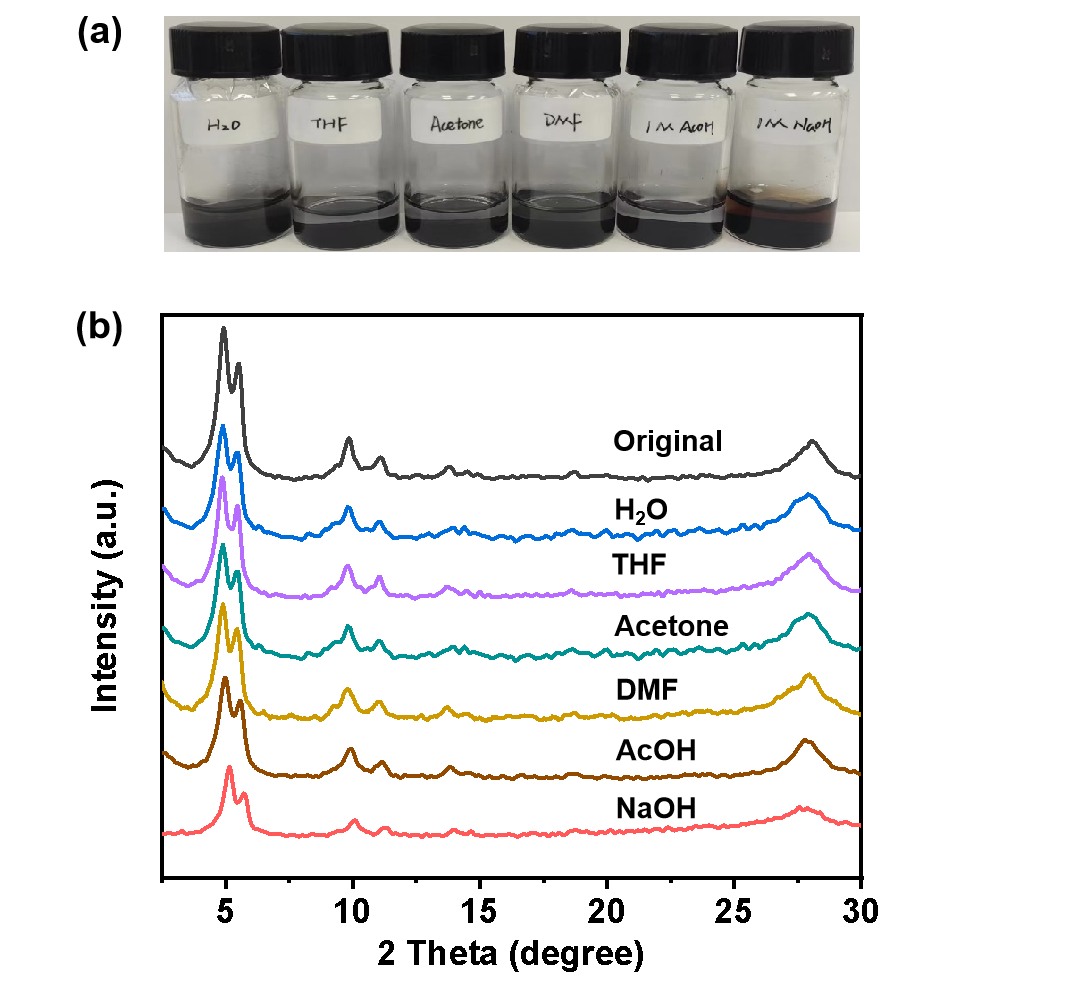


**Figure S20.** (a) Images and (b) PXRD patterns of Cu-TTPQ after immersing in various solvents for five days (PXRD patterns were collected on a DX-27 mini 400 W X-Ray diffractometer).

**Section 12. Thermal Stability Test**


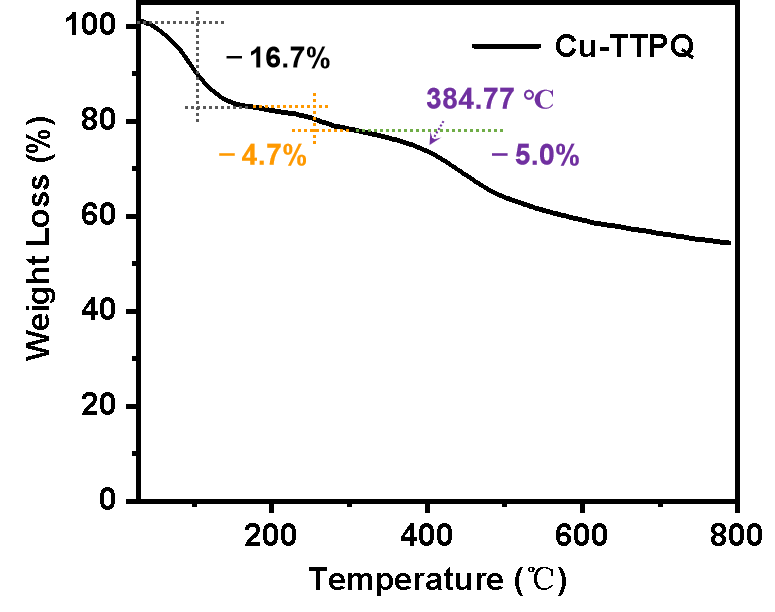


**Figure S21.** TG curve of the Cu-TTPQ under nitrogen atmosphere.

The removal of solvents of Cu-TTPQ are observed through two consecutive mass losses of approximately 17% (6 H_2_O) and 5% (1 DMF) could be attributed to the trapped H_2_O and DMF solvent molecules in Cu-TTPQ, respectively. TGA curve exhibits a mass loss of about 5% without solvent loss in skeleton at 385 ℃, indicating a good thermal stability.

**Section 13. N_2_ Sorption Isotherms and Pore Size Distribution**

**
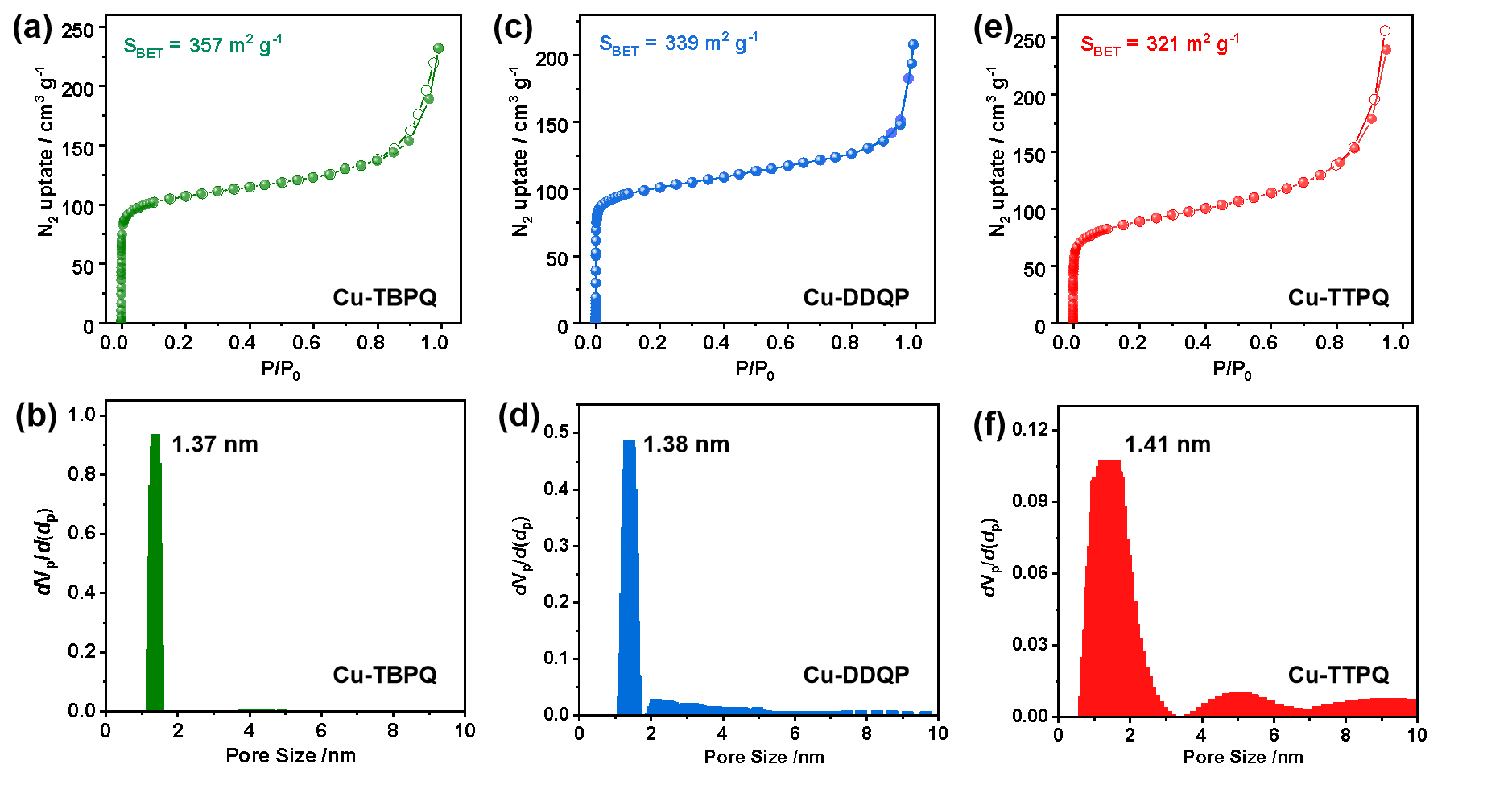
Figure S22.** N_2_ adsorption desorption isotherms and pore sizes of (a and b) Cu-TBPQ (green), (c and d) Cu-DDQP (blue) and (e and f) Cu-TTPQ (red) powder at 77 K.

**Section 14. Electrical Conductivity Measurements**


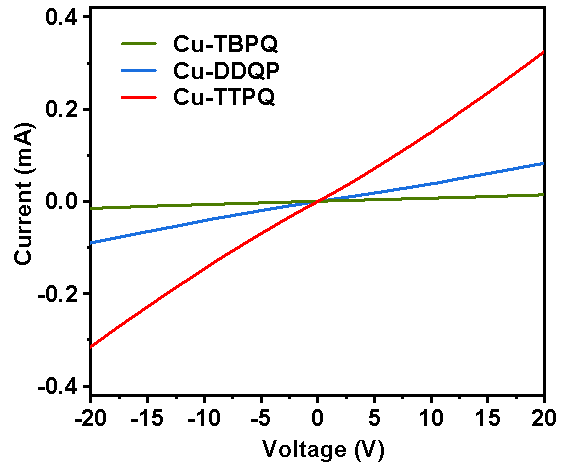


**Figure S23.** *I-V* curves of Cu-TBPQ (green line), Cu-DDQP (blue line) and Cu-TTPQ (red line) pellets at room temperature.

Table **S2**. Electrical conductivity test parameters for Cu-TBPQ, Cu-DDQP and Cu-TTPQ pellets.

|  | **Area (cm^2^)** | **Thickness (mm)** | **Resistance (KΩ)** | **Conductivity (S cm^−1^)** |
| --- | --- | --- | --- | --- |
| Cu-TBPQ | 0.79 | 0.296 | 952.39 | 6.1 × 10^-7^ |
| Cu-DDQP | 0.79 | 0.343 | 186.92 | 2.3 × 10^-6^ |
| Cu-TTPQ | 0.79 | 0.266 | 61.35 | 5.6 × 10^-6^ |

**Section 15. LUMO-HOMO Energy Levels of the Cylcodehydrogenated Ligands**


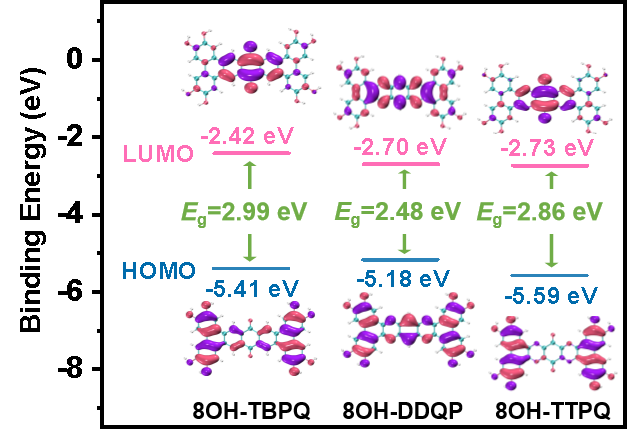


**Figure S24.** LUMO-HOMO gap of the cyclodehydrogenated 8OH-TBPQ, 8OH-DDQP and 8OH-TTPQ.


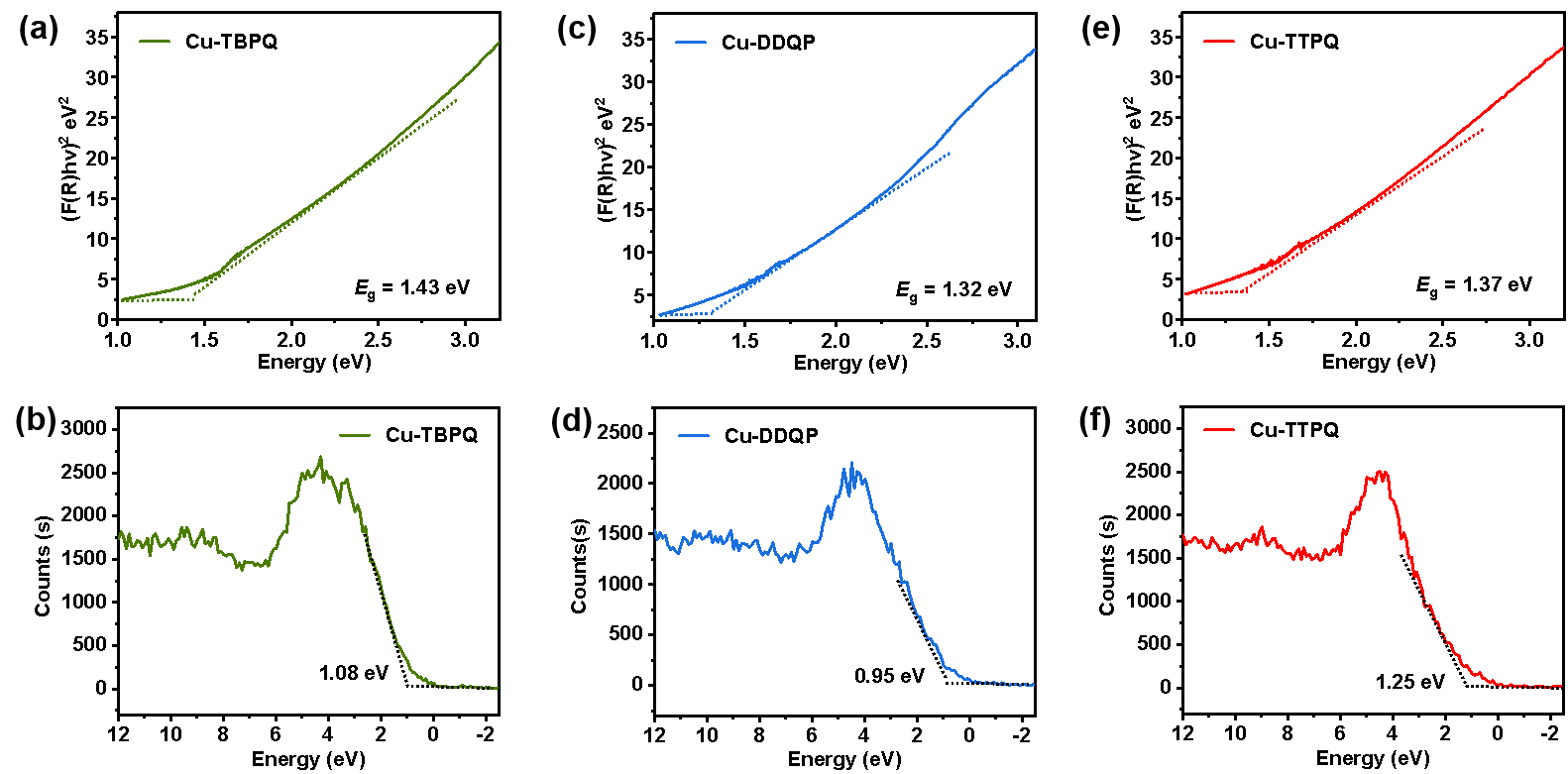


**Figure S25.** Tauc-plots derived from UV-vis-NIR and VB-XPS spectra of (a, b) Cu-TBPQ, (c, d) Cu-DDQP and (e, f) Cu-TTPQ.

**Table S3**. Experimental LUMO-HOMO gap of Cu-TBPQ, Cu-DDQP and Cu-TTPQ.

|  | **LUMO^a^ (eV)** | **HOMO^b^ (eV)** | ***E*_g_^c^ (eV)** |
| --- | --- | --- | --- |
| Cu-TBPQ | -3.85 | -5.28 | 1.43 |
| Cu-DDQP | -3.83 | -5.15 | 1.32 |
| Cu-TTPQ | -4.08 | -5.45 | 1.37 |

^a^HOMO = -(*E*_SHE_-*Φ*), *E*_SHE_ of Cu-TBPQ, Cu-DDQP and Cu-TTPQ were measured by VB-XPS spectra (corresponding bonding energy of standard hydrogen electrode) in Figures S25b, S25d and S25f, *Φ* = -4.2 eV.

^b^LUMO = *E*_g_+HOMO.

^c^The band gap (*E*_g_) of Cu-TBPQ, Cu-DDQP and Cu-TTPQ were measured according to Tauc-plot derived from UV-vis absorption in Figures S25a, S25c and S25e.

**Section 16. Electrochemical Properties**


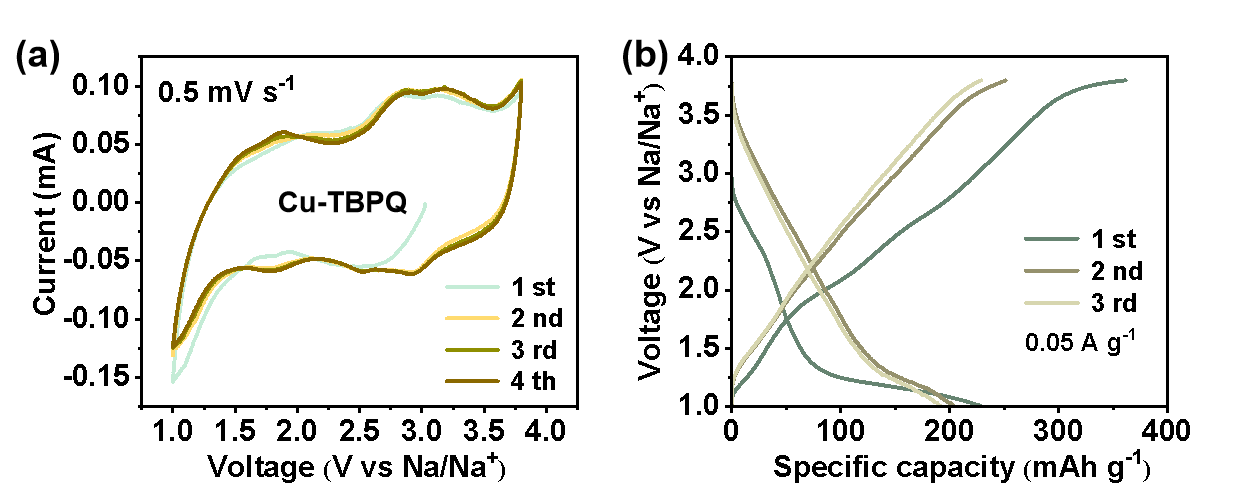


**Figure S26.** The electrochemical performance of Cu-TBPQ electrode: (a) CV curves at 0.5 mV s^−1^ and (b) GCD profiles at 0.05 A g^−1^.


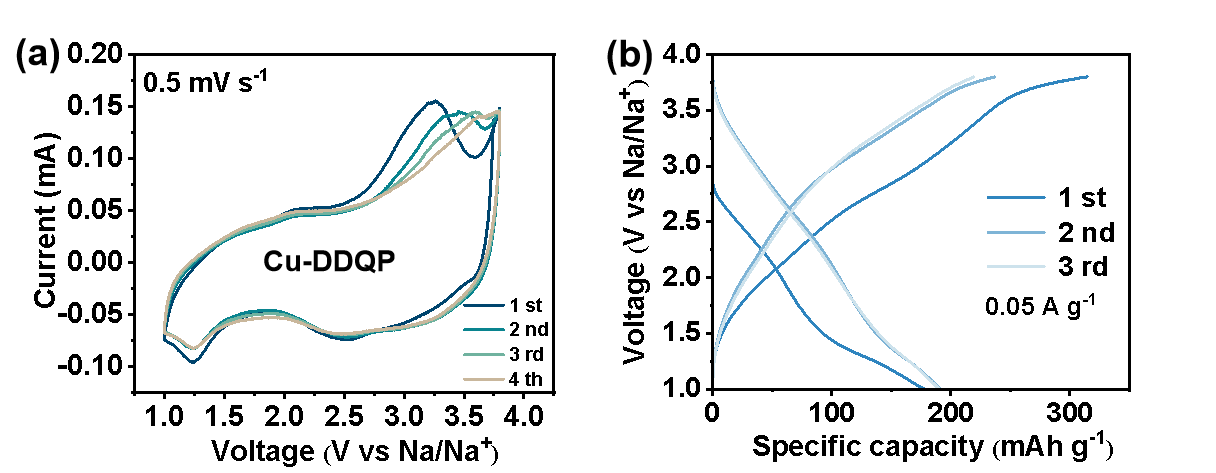


**Figure S27.** The electrochemical performance of Cu-DDQP electrode: (a) CV curves at 0.5 mV s^−1^ and (b) GCD profiles at 0.05 A g^−1^.

**
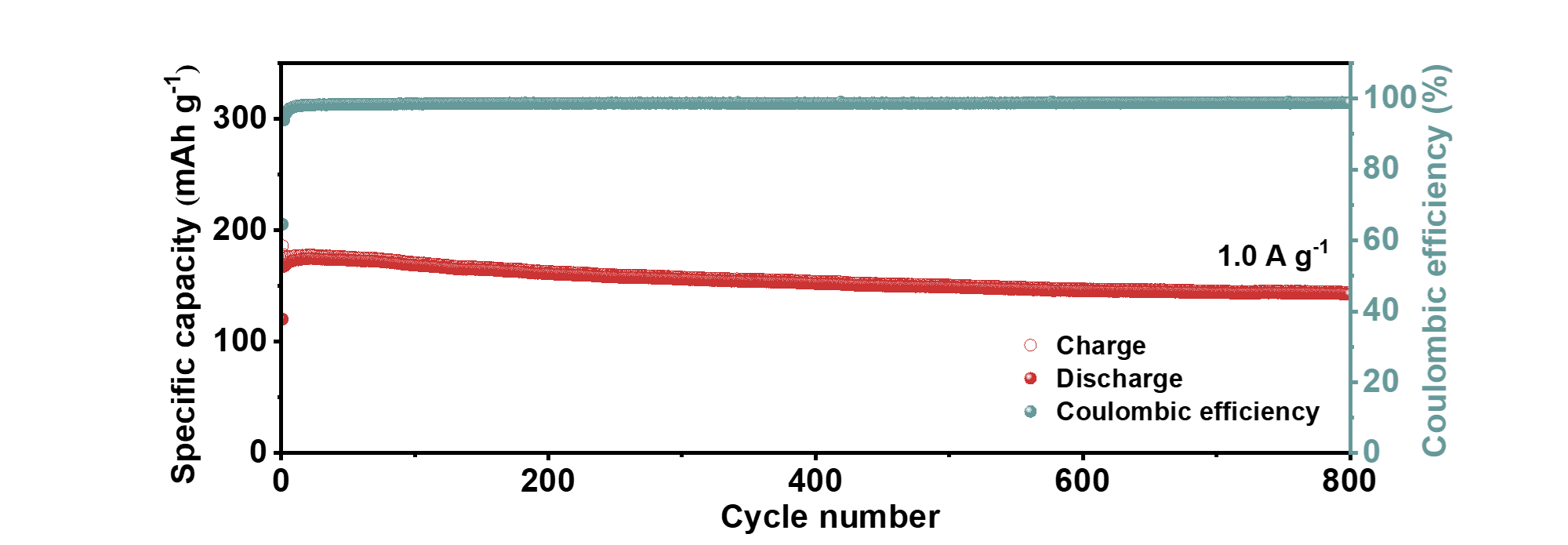
**

**Figure S28.** Cycling performance of Cu-TTPQ electrode at 1.0 A g^−1^.


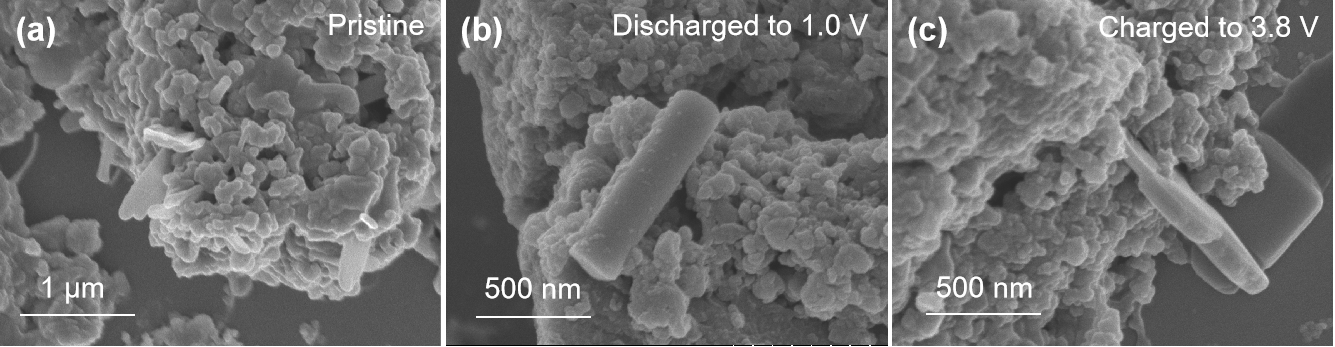


**Figure S29.** SEM images of the Cu-TTPQ cathode in the (a) pristine, (b) discharged and (c) charged states. The particle size and shape exhibit no significant changes after charging and discharging.

**Table S4.** Capacitance of Cu-TBPQ, Cu-DDQP and Cu-TTPQ at different current densities.

|  | Num. of cycles |  | 0.1  A g^−1^ | 0.2  A g^−1^ | 0.5  A g^−1^ | 1.0  A g^−1^ | 2.0  A g^−1^ |
| --- | --- | --- | --- | --- | --- | --- | --- |
| Cu-TBPQ | 1-50 | mAh g^−1^ | 155.5 | 131.9 | 105.1 | 78.8 | 49.8 |
|  | 51-90 | mAh g^−1^ | 136.9 | 125.7 | 103.1 | 79.0 |  |
| Cu-DDQP | 1-50 | mAh g^−1^ | 165.7 | 149.2 | 121.6 | 100.9 | 80.8 |
|  | 51-90 | mAh g^−1^ | 173.8 | 146.7 | 119.1 | 99.9 |  |
| Cu-TTPQ | 1-50 | mAh g^−1^ | 216.7 | 199.0 | 162.1 | 134.6 | 107.8 |
|  | 51-90 | mAh g^−1^ | 218.9 | 195.6 | 158.8 | 133.20 |  |

**Table S5.** Electrochemical performance of reported 2D *c*-MOFs as electrodes for SIBs.

| **Electrode** | **Type** | **Operating voltage (V)** | **Low-rate capacity (mAh g^-1^) (Current density, mA g^-1^)** | **Cycle**  **Number**  **(Current density, mA g^-1^)** | **Capacity retention**  **(%)** | **Ref.** |
| --- | --- | --- | --- | --- | --- | --- |
| Cu-TTPQ | Cathodes | **1.0-3.8** | **215** **(50)** | **1800 (5000)** | **99** | ***This work*** |
| Cu-TBPQ |  | 1.0-3.8 | 190 (50) | 100 (200) | 76 |  |
| Cu-DDQP |  | 1.0-3.8 | 191 (50) | 100 (200) | 93 |  |
| Cu-HHTP |  | 1.0-3.6 | 175 (100) | 600 (1000) | 80 | [5] |
| Zn-HHTP |  | 1.0-3.5 | 150 (100) | 5000 (1000) | 70 | [5] |
| Cu-TBA |  | 1.0-3.5 | 154 (50) | 3000 (1000) | 99 | [6] |
| Cu-TABQ  (CCP) |  | 1.0-3.8 | 305 (100) | 2000 (5000) | 100 | [7] |
| Co-HAB | Anodes | 0.5-3.0 | 291 (50) | 150 (4000) | 95 | [8] |
| HATN-OCu |  | 0.01-3.0 | 500 (100) | 1000 (2000) | 58 | [9] |
| HATN-SCu |  | 0.01-3.0 | 231 (100) | 1000 (2000) | 100 | [9] |

**Section 17.** **Reaction and Diffusion Kinetics**


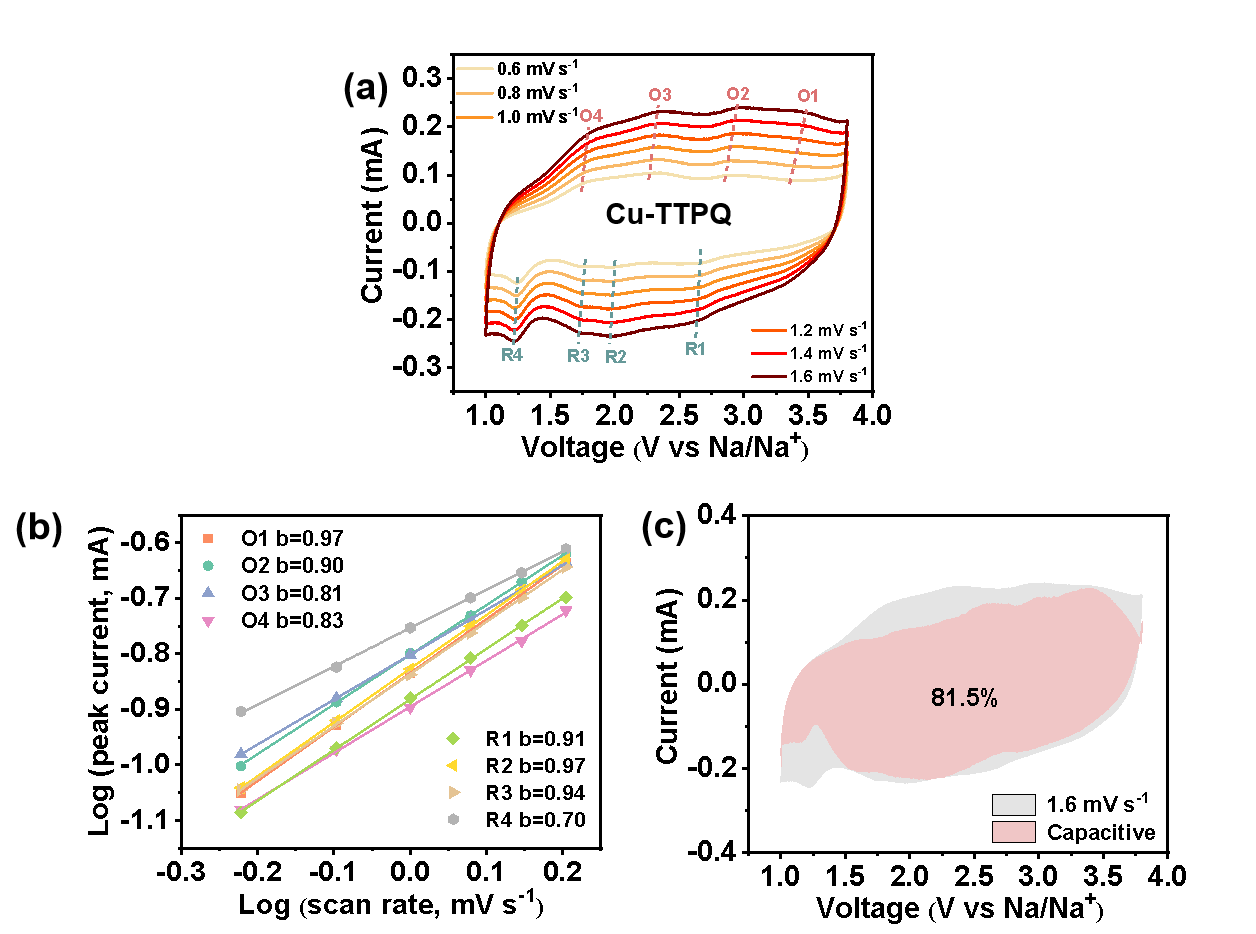


**Figure S30.** Electrode process kinetics of Cu-TTPQ. (a) CV curves recorded at different scan rates, (b) the linear relationship between log(i) and log(v) and (c) capacitive and diffusion-controlled contribution at 1.6 mV s^−1^.


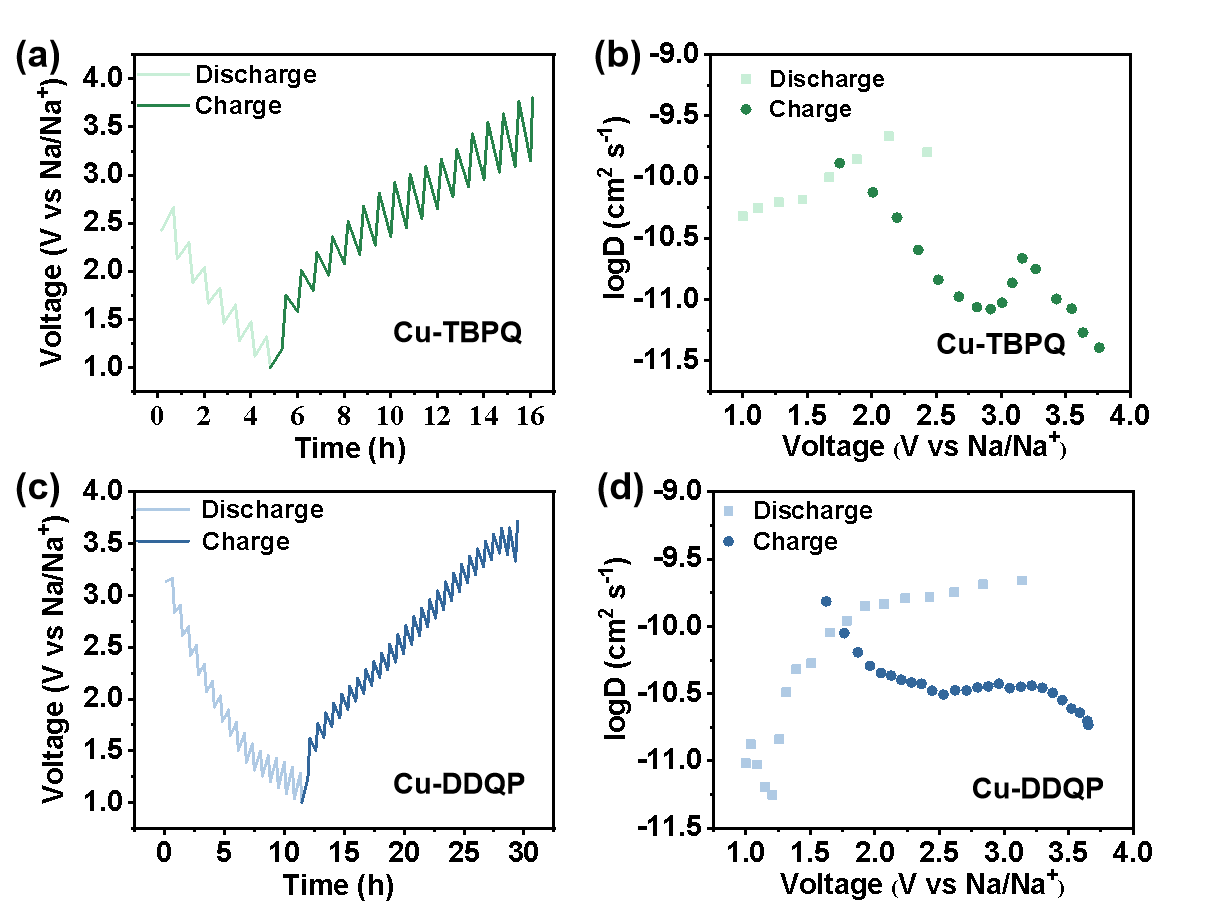


**Figure S31.** GITT curves and calculated diffusion coefficient of (a, b) Cu-TBPQ and (c, d) Cu-DDQP.


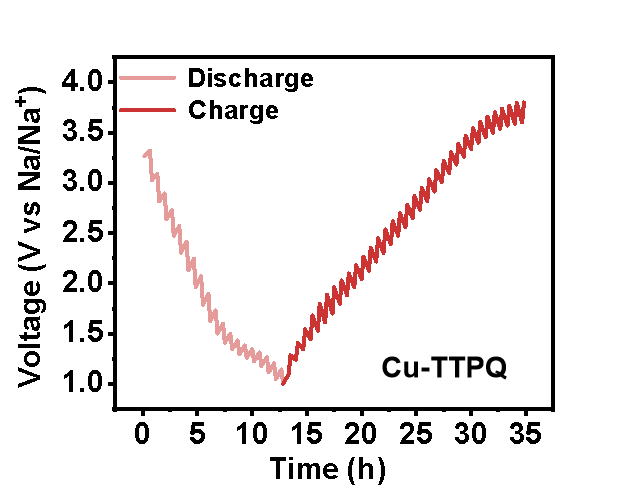


**Figure S32.** GITT curve of Cu-TTPQ.

**Section 18. Structural** **Calculations and Optimizations of Cu-TTPQ-*n*Na**

**
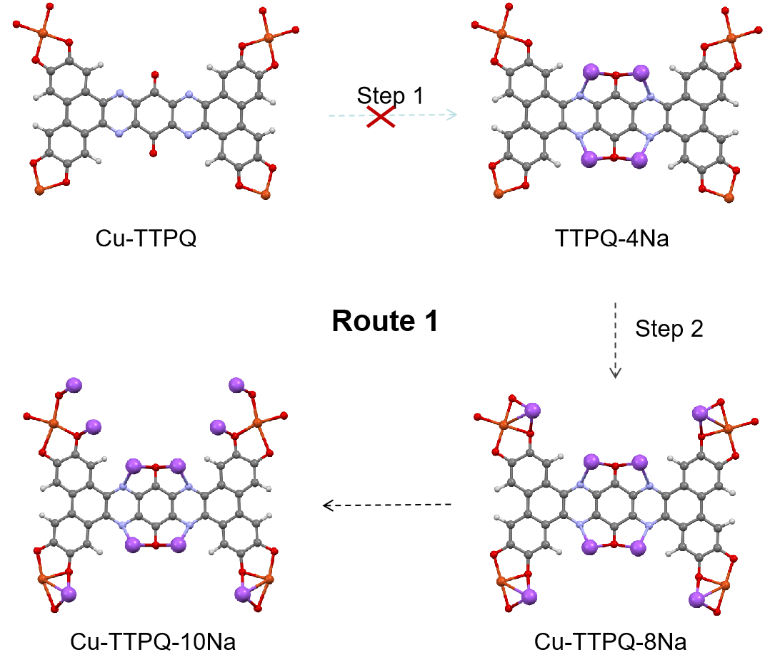
**

**Figure S33.** The structure evolution of ions storage mechanism for Na^+^ preferentially bonding to ligands.

**
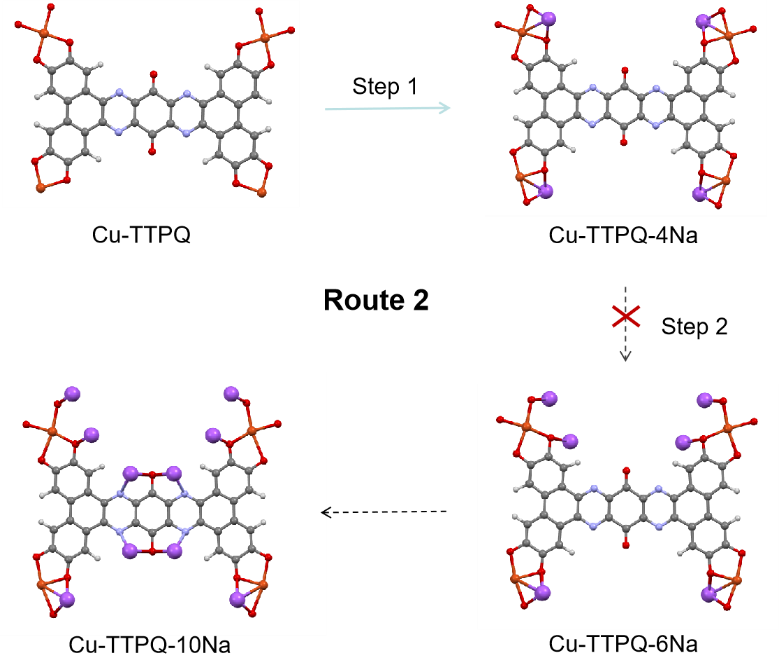
**

**Figure S34.** The structure evolution of ions storage mechanism for Na^+^ preferentially bonding to [CuO_4_].

**Table S6.** The calculated energy corresponding to the cations stored in Cu-TTPQ-*n*Na.

|  | Crystal cell | Adsorption energy (*E*_adv_, eV) | Redox potential (V) |
| --- | --- | --- | --- |
| Cu-TTPQ | *a* = 19.14600 Å, *b* = 19.14606 Å, *c* = 3.27882 Å  *α =* 90.0000°, *β* = 90.0000°, *γ* =110.3507° | / | / |
| Cu-TTPQ-4Na | *a* = 19.09577 Å, *b* = 19.10425 Å, *c* = 3.48557 Å  *α =* 90.0000°, *β* = 90.0000°, *γ* =109.9797° | -**2.23** | **2.23** |
| Cu-TTPQ-6Na | *a* = 19.20091 Å, *b* = 19.19992 Å, *c* = 3.49170 Å  *α =* 90.0000°, *β* = 90.0000°, *γ* =108.8014 | -1.63 | 0.43 |
| TTPQ-4Na | *a* = 19.14262 Å, *b* = 19.12317 Å, *c* = 3.35395 Å  *α* = 90.0000°, *β* = 90.0000°, *γ* =109.1234° | -2.17 | / |
| Cu-TTPQ-8Na | *a* = 1917946 Å, *b* = 19.18005 Å, *c* = 3.52702 Å  *α =* 90.0000°, *β* = 90.0000°, *γ* =109.7773° | -**2.02** | **1.80** |
| Cu-TTPQ-10Na | *a* = 19.25624 Å, *b* = 19.25434 Å, *c* = 3.53800 Å  *α* = 90.0000°, *β* = 90.0000°, *γ* =107.8921° | -1.63 | 0.07 |

**Section 19. Supporting References**

1. T. Chen, J. H. Dou, L. Yang, C. Sun, J. J. Oppenheim, J. Li, M. Dincă, *J. Am. Chem. Soc*. **2022**, *144*, 5583–5593.
2. A. Shah, V. K. Vishwakarma, N. Lhouvum, A. A. Sudhakar, P. Kumar, A. K. Srivastava, F. Dubois, T. Chomchok, N. Chattham, D. P. Singh, L. Chen, *Journal Mol. Liq.* **2024**, *393*, 123535.
3. J. Liu, Y. Zhou, G. Xing, M. Qi, Z. Tang, O. Terasaki, L. Chen, *Adv. Funct. Mater*. **2024**, *34*, 2312636.
4. X. Sun, X. Yan, K. Song, T. Zhang, Z. Yang, X. Su, W. Chen, L. Chen, *Chin. J. Chem*. **2023**, *41*, 1691–1696.
5. Y. Chen, Q. Zhu, K. Fan, Y. Gu, M. Sun, Z. Li, C. Zhang, Y. Wu, Q. Wang, S. Xu, J. Ma, C. Wang, W. Hu, *Angew. Chem. Int. Ed*. **2021**, *60*, 18769‒18776.
6. M. Qi, L. Cheng, H. G. Wang, F. Cui, Q. Yang, L. Chen, *Adv. Mater*. **2024**, *36*, 2401878.
7. K. Fan, C. Fu, Y. Chen, C. Zhang, G. Zhang, L. Guan, M. Mao, J. Ma, W. Hu, C. Wang, *Adv. Sci.* **2022**, *10*, 2205760.
8. J. Park, M. Lee, D. Feng, Z. Huang, A. C. Hinckley, A. Yakovenko, X. Zou, Y. Cui, Z. Bao, *J. Am. Chem. Soc*. **2018**, *140*, 10315‒10323.
9. B. Wang, J. Li, M. Ye, Y. Zhang, Y. Tang, X. Hu, J. He, C. C. Li, *Adv. Funct. Mater*. **2022**, *32*, 2112072.
